# Integrative machine learning approach for identification of new molecular scaffold and prediction of inhibition responses in cancer cells using multi-omics data

**DOI:** 10.1093/bfgp/elaf006

**Published:** 2025-04-19

**Authors:** Aman Chandra Kaushik, Shubham Krushna Talware, Mohammad Imran Siddiqi

**Affiliations:** Division of Biochemistry and Structural Biology, CSIR-Central Drug Research Institute, Jankipuram Extension, Sitapur Road, Lucknow, Uttar Pradesh 226031, India; Department of Technology Dissemination and Computational Biology Division, CSIR-Central Institute of Medicinal and Aromatic Plants, P. O. CIMAP, Kukrail Road, Lucknow 226015, India; IndiaAcademy of Scientific and Innovative Research (AcSIR), Ghaziabad 201002, India; Division of Biochemistry and Structural Biology, CSIR-Central Drug Research Institute, Jankipuram Extension, Sitapur Road, Lucknow, Uttar Pradesh 226031, India; Division of Biochemistry and Structural Biology, CSIR-Central Drug Research Institute, Jankipuram Extension, Sitapur Road, Lucknow, Uttar Pradesh 226031, India

**Keywords:** cancer, molecular scaffold, scRNA-Seq, Idasanutlin, machine learning

## Abstract

MDM2 (Mouse Double Minute 2), a fundamental governor of the p53 tumor suppressor pathway, has garnered significant attention as a favorable target for cancer therapy. Recent years have witnessed the development and synthesis of potent MDM2 inhibitors. Despite the fact that numerous MDM2 inhibitors and degraders have been assessed in clinical studies for various human cancers, no FDA-approved drug targeting MDM2 is presently available in the market. Researchers have investigated the effects of various drugs, which are involved in cancer therapies with known mechanisms, on well-characterized cancer cell lines. The prediction of drug inhibition responses becomes crucial to enhance the effectiveness and personalization of cancer treatments. Such findings can provide new perceptions aimed at designing new drugs for targeted cancer therapies. In our current insilico work, a robust response was observed for Idasanutlin in cancer cell lines, indicating the drug’s significant impact on gene expression. We also identified transcriptional response signatures, which were informative about the drug’s mechanism of action and potential clinical application. Further, we applied a similarity search approach for the identification of potential lead compounds from the ChEMBL database and validated them by molecular docking and dynamics studies. The study highlights the potential of incorporating machine learning with omics and single-cell RNA-seq data for predicting drug responses in cancer cells. Our findings could provide valuable insights for improving cancer treatment in the future, particularly in developing effective therapies.

## Graphical Abstract

**Graphical Abstract:**
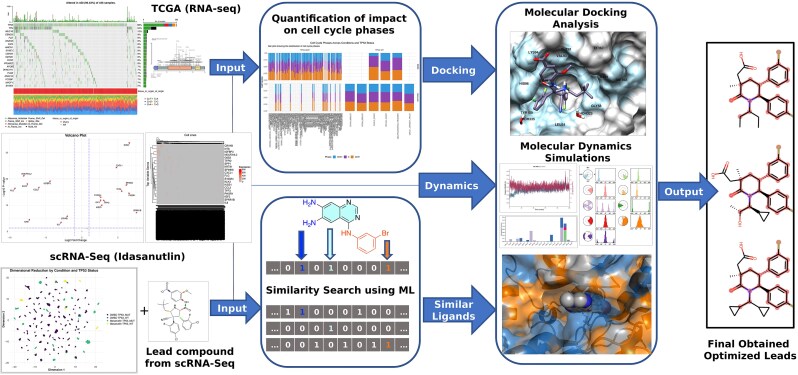

The study investigates the effects of Idasanutlin on well-characterized cancer cell lines, targeting eight cancer therapies with known mechanisms. The researchers identified a robust response in TP53 WT cell lines, highlighting the potential of this drug in cancer therapy. This study identified transcriptional response signatures that provide valuable insight into the drug’s potential clinical application and molecular mechanisms. Mutational effects in significant cancers, including prostate, ovarian cancer, and kidney, were noted, emphasizing the importance of understanding molecular pathways. Integrating machine learning with multi-omics and single-cell RNA-seq data offer promising avenues for predicting drug responses in cancer cells and improving cancer therapy.

## Introduction

Cancer remains a significant health burden worldwide, and identifying effective therapies is crucial in reducing its impact [1, 2]. The heterogenic nature of the disease makes it more difficult for clinicians and scientists to implement targeted therapies for cancer patients [3]. Also, the anticancer medications have varied degrees of efficacy, on patients suffering from same cancer sub-types, due to the genetic variability among them. Hence, the conventional ‘blanket’ approach can never accommodate the vast differences among the individuals undergoing treatment therapy for cancer. This phenomenon led to the advent of precision medicine which alters the drug and dose requirement according to the individual’s genetic profile rendering the treatment more patient-specific [4].

Precision oncology aims at delivering better health outcomes and cost-effective treatment for patients with different cancer types and sub-types [5, 6]. Considering diverse factors including tumor heterogeneity, driver oncogenic genes, and aberrant molecular/genetic alterations, precision oncology can provide better recommendations for tailored therapy decisions. In 2017, the nod for Pembrolizumab by FDA marked the first example of drug approval based on precision oncology based on 15 types of cancer [7]. Similarly, Larotrectinib hints at another potential break-through in future for targeting tropomyosin receptor kinase gene fusion in a variety of cancers [7, 8]. These examples insinuate on the fact that the upcoming years will show a dynamic shift in the course of drug discovery and development from the conventional approach to a more patient-specific and genetic profile driven approach.

‘Omics’ data addresses the essential requirements for personalized medicine by encompassing knowledge regarding the complex biological processes and the inter-relationship between biological micro and macromolecules [9]. Recent advances in genomic technologies have permitted the analysis of various molecular levels of biological systems, including genomics, transcriptomics, proteomics, and metabolomics [10]. These molecular profiles provide a comprehensive view of the cellular and molecular processes underlying biological systems. However, analyzing these profiles individually often limits our understanding of the complex interactions that occur within biological systems. Compared to single-layer omics analysis, multiple-omics data offers a more comprehensive view of the molecular processes associated with biological systems [11, 12]. This method is richer in information density, more robust to reproduction, and enables researchers to identify previously undetected molecular alternations associated with various diseases.

By amalgamating genetic mutation, gene expression, and pathway activation data, researchers can ascertain the complex relationships and mechanisms underlying cancer development and progression. Integrating multiple-omics data can provide insight into the molecular mechanisms underlying different cancers and identify novel potential therapeutic targets for the same [13–16]. The utilization of publicly available pharmacogenomics data allows for a more precise and personalized treatment strategy that targets the unique molecular alterations associated with specific cancers [14, 15].

Recent advances in computational methods have greatly enhanced the predictive power of multi-omics data. Machine learning and deep learning techniques have been employed to analyze complex multi-omics datasets and resulted in improved accuracy in drug response prediction [17, 18]. Methods like graph neural networks along with autoencoders help to incorporate heterogeneous data, facilitating robust modeling for drug responses [19, 20]. Such models help in identifying biomarkers and molecular signatures which predict how individual patients respond to specific therapies, paving the way for personalized oncology [21]. Despite these advancements, several challenges hinder the full exploration of multi-omics data’s potential in predicting drug responses.

One of the major challenges is technical complexity to collate and interpret the vast and heterogeneous datasets, taking the variability existing between and within tumors into consideration [16]. Hence, the models must accommodate for dynamic nature of evolution of this disease and the resulting resistance development to the treatment. Moreover, missing or inconsistent data as well as the presence of class imbalance along with factors such as batch effects may also contribute to poor performances of the established models [22]. Advance computational tools are essential to handle these complex data and should be refined from time to time to combat with lacking interpretability and reproducibility of such models [14, 16, 23]. The clinical implementation of such models also suffers from significant hurdles due to the time-consuming and resource-intensive process of validation in diverse patient populations, which is necessary to ensure the accuracy and reliability of multi-omics models [24]. The complexity of the process is substantially increased when the ethical, social, and current status of the regulatory framework as well as health infrastructure taken into consideration.

In recent years, exploring the use of drugs that target specific molecular pathways in cancer cells has escalated [17]. In this study, we tried to incorporate machine learning methods to predict drug responses against cancer cell lines using multi-omics data. The effects of Idasanutlin on well-characterized cancer cell lines, targeting cancer therapies with known mechanisms, have been investigated in this study followed by the identification of new scaffolds based on the similarity search approach as shown in the graphical abstract.

## Materials and methods

### Data collection

The Cancer Genome Atlas (https://www.cancer.gov/tcga) platform was used to get data on somatic mutations, gene expressions, and clinical information of various cancers utilized for our insilico study. Using the R package ‘TCGAbiolinks’ [25], the data was obtained from the Genomic Data Commons Data Portal (https://portal.gdc.cancer.gov/) in October 2022. The dataset called MuTect2 [26] was used to fetch variant aggregation and masking since it comprehended comparatively more gene alternations than others. A total of 436 samples of ovarian cancer were considered for our study, where the clinical and mutational data were selected for further analysis.

### Drug screening from single-cell RNA-seq data

**Figure 1:**
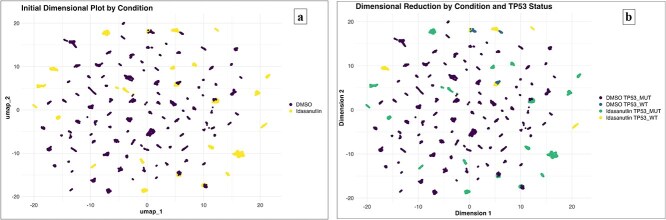
Plot demonstrating the gene expression profiles of cells with varying TP53 statuses (wild-type *versus* mutant) in response to Idasanutlin treatment. (**a**) UMAP visualization of cells treated with DMSO control (violet) and Idasanutlin (yellow) against a diverse set of cell lines before dimensionality reduction. Two distinct clusters correspond to the two experimental conditions. (**b**) UMAP visualization of cells treated with DMSO and Idasanutlin against a diverse set of cell lines after dimensionality reduction.

The analysis utilized a number of software packages to perform various tasks. Seurat v4.3.0 [27–32] was used for scRNA-seq analysis. All data reported in this manuscript with respect to scRNA-sequencing, drug sensitivity, and other cell line features, are available on the Figshare Dataset [33]. To differentiate biologically significant alterations in gene expression with the perspective of drugs, we utilized RNA-seq and scRNA-seq data; the detailed information is shown in supplementary data. This study includes UMAP plotting and volcano plot analysis discussed in the result section below (Fig. 1 and Fig. S1).

### Similarity search for tumor suppressor p53/oncoprotein MDM2 analysis using machine learning

RG7388 (Idasanutlin) was reported for the treatment of infected cancer cell lines, triggering an up-regulation of MDM2 in response to elevated p53 levels, by a negative feedback mechanism [34]. It was observed that Idasanutlin has been found to significantly impact the gene expression of cancer cells [35, 36]. Therefore, we used Idasanutlin as a reference for the similarity search approach. Using a Python script, we incorporated ligand-based virtual screening on the compounds retrieved from the ChEMBL database [37]. We examined the retrieved data for compounds pre-processing, to filter for bioactivity against the tumor suppressor p53/oncoprotein MDM2. The filtered compounds were further subjected to data refinement for the optimal pharmacokinetic profiles. Further, for the curated compounds fingerprints were computed, following which they were subjected to clustering on the maximum common substructure (MCS) criteria *i.e.* clustering compounds based on the most common scaffold/backbone present across the majority of compounds. The complete refinement steps along with a detailed statistical analysis of these parameters are explained in supplementary data.

Random Forest (RF) [37], Support Vector Machine (SVM) [38], and Artificial Neural Network (ANN) [39] were the three ML models applied to predict the compounds that were retrieved from the largest cluster. The compounds with a pIC50 of > = 6.0 were labelled as active and were subjected to molecular encoding using the MACCS and Morgan fingerprint method. Further, the performances of all ML models were analyzed and compared (as shown in Table 1). The compounds predicted by the best performing model were then validated by structure-based methods (molecular docking and dynamics) ensuring the robustness of our methodology for screening potential MDM2 inhibitors.

**Table 1:** Performance of all three models (RF, SVM, and ANN) and their taken time.

**Models**	**RF**	**SVM**	**ANN**
**Mean accuracy (Mean)**	0.90	0.89	0.88
**Mean sensitivity (Mean)**	0.91	0.89	0.88
**Mean specificity (Mean)**	0.90	0.89	0.88
**Mean AUC (Mean)**	0.95	0.95	0.94
**Mean accuracy (SD)**	0.01	0.02	0.01
**Mean sensitivity (SD)**	0.04	0.04	0.02
**Mean specificity (SD)**	0.03	0.04	0.01
**Mean AUC (SD)**	0.00	0.01	0.02
**Time taken (seconds)**	0.76	0.21	1.41

### Molecular docking study

To get insights into the disruptions of the MDM2-p53 complex system that is proposed to be caused by the screened compounds, we performed a docking analysis of the 80 compounds (which was obtained from the machine learning approach) against the co-crystallized structure of MDM2 (PDB ID: 4ERF). The docking study was carried out with Schrodinger’s (Schrödinger Release 2023–2: LigPrep, Schrödinger, LLC, New York, NY, 2021.) in-built GLIDE module [40]. At first, the compounds were optimized with the LigPrep module of Schrodinger, and simultaneously, the Protein Preparation wizard [41] was utilized for rendering the protein available for the docking study. Then, a receptor grid was generated around the co-crystallized ligand so as to confine the study within the orientation of the ligand itself. It was followed by redocking of the bound ligand extracted from MDM2 (PDB ID: 4ERF) to validate the reliability of the study. All the compounds were initially subjected to SP and further XP modes of docking, penalizing the mismatched orientation and enrichment of the analysis. The best compounds (along with compound A: Idasanutlin) were shortlisted on the basis of docking score, structural orientation, and specific interactions pertaining to the bound ligand (AM-8553).

### Molecular dynamics simulation study

The molecular dynamics (MD) simulation study was implemented and analyzed using the Desmond package v31023 [42–44]. The MD simulation of the MDM2 complex with the top 13 screened compounds (compound A to compound M) was carried out using the Desmond Schrodinger package. Each protein-ligand complex underwent a 100 nanosecond (ns) simulation, resulting in a total simulation time of 1300 ns. The details of system preparation for the MD simulation study are mentioned in the supplementary data. The plots of RMSD (root mean square deviation) and RMSF (root mean square fluctuation) of the complexes, for each compound were evaluated throughout the simulation trajectory using the OPLS2005 (Optimized Potentials for Liquid Simulations) force field [44].

## Results

### Potential drugs for *TP53* mutation

After performing an initial analysis of IC50 values for different cancer cell lines and their transcriptomic profiles, we observed the mutational pattern of TP53 in ovarian cancer. It was noted that this pattern was highly scattered, making it challenging to identify potential target mutations. Hence, we compared the response of various drugs against these mutated and non-mutated cell lines, identifying Idasanutlin as a potential therapeutic candidate. Further, we examined the IC50 drug sensitivity measure in scRNA-Seq data to identify prospective therapeutic targets for all cancer cell lines.

### Single-cell expression profiles

For the scRNA sequencing analysis, 24 different cancer cell lines including DKMG_CENTRAL_NERVOUS_SYSTEM, BT549_BREAST, SQ1_LUNG, IALM_LUNG, CCFSTTG1_CENTRAL_NERVOUS_SYSTEM, NCIH2347_LUNG, RERFLCAD1_LUNG, BICR6_UPPER_AERODIGESTIVE_TRACT, LS1034_LARGE_INTESTINE, UMUC1_URINARY_TRACT, COV434_OVARY, SKMEL3_SKIN, LNCAPCLONEFGC_PROSTATE, BT474_BREAST, BICR31_UPPER_AERODIGESTIVE_TRACT, SH10TC_STOMACH, RCM1_LARGE_INTESTINE, SKMEL2_SKIN, RCC10RGB_KIDNEY, NCIH226_LUNG, TEN_ENDOMETRIUM, SNU1079_BILIARY_TRACT, CAOV3_OVARY, and COLO680N_OESOPHAGUS were used. Detailed information about the source, tumor type, and subtypes, status information for associated disease conditions, and mutation patterns regarding these cell lines can be found on the DepMap Portal, which is an open-source research domain for visualization, analysis, and exploring cancer dependencies [45]. All the data reported in the manuscript for scRNA-sequencing, drug sensitivity, and other cell line features, are available on the Figshare Dataset [33].

We generated 2D representations of the single-cell expression profiles using Seurat v4.3.0 [27]. The single-cell counts data underwent initial normalization and log transformation using the NormalizeData function. Subsequently, cell-wise normalization was performed using the ScaleData function. We employed the FindVariableGenes function to select the top 5000 genes with the highest variability, employing the ‘vst’ selection method. The RunPCA function was used to perform Principal Component Analysis (PCA), and the top 2 N principal components – where N is the number of cell lines in the pool was retained. The RunTSNE function was then employed to create the t-SNE embeddings for the calculated principal components. For UMAP embeddings, we utilized the RunUMAP Seurat function, applying default parameters for ‘min.dist’ (step-wise details are mentioned in the supplementary data).

The resulting 2D UMAP representations enabled the analysis of individual cell expression profiles within each cancer cell line, facilitating the identification of potential clusters exhibiting similar gene expression patterns. The UMAP plots demonstrate that within the same cancer cell lines, cells show variability by exhibiting different and independent clusters. This variation arises due to differing drug conditions, mutation status of TP53, or other inherent differences among cell lines. Here, understanding the heterogeneous nature of tumors is essential as it advocates for the developing resistance to the existing drug therapies among subpopulations of similar cancer subtypes. Hence, this approach offers valuable insights into the heterogeneity and developing mutations of cancer cell populations enabling the generation of personalized medications and tailored therapies for patients in cancer management. The results of PCA for dimensionality reduction and UMAP analyses are discussed below.

**Figure 2:**
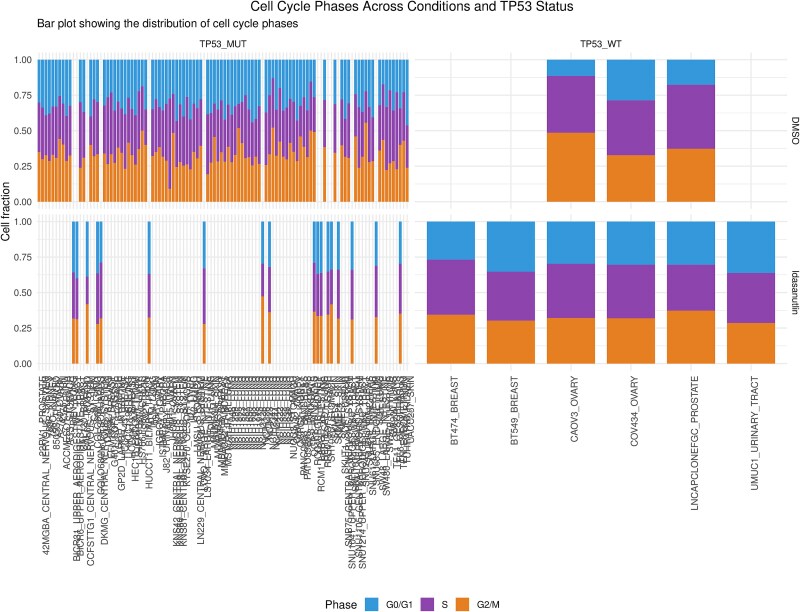
Cell-cycle phase distribution, categorized by cell line and TP53 statuses, depicted as a fraction of cells for each condition. The plot represents the variation across different cell cycle phases in response to Idasanutlin in wild-type and mutant cell lines.

Idasanutlin: The UMAP embedding was generated and colored using the ‘singlet_ID’ (assigned to an individual cell; since during the single-cell experiments, two or more cells may be captured in a single droplet) metadata column, which indicates the most probable cell line for each cell, based on its SNP profile. The ‘singlet_ID’ helps to maintain the integrity of cell-type-specific gene expression analysis. The UMAP representation indicates two clusters with respect to the DMSO control (colored in violet) and Idasanutlin treatment (colored in yellow) across a pool of cell lines (Fig. 1). Notably, only TP53 wild-type cell lines exhibited a significant transcriptional response to Idasanutlin. Moreover, when visualizing the UMAP embedding based on the cell-cycle phase, it became evident that the transcriptional heterogeneity in TP53 mutant cell lines was also driven by the cell-cycle dynamics. The fraction of cells from each cell line in different cell-cycle phases varied depending on the treatment condition and TP53 status as shown in Fig. 2. Together, Figs. 1 and 2 provide a clear depiction of the clustering patterns based on TP53 status and cell-cycle phases under Idasanutlin treatment conditions, which is crucial for understanding treatment response variability across genetic backgrounds.

**Figure 3:**
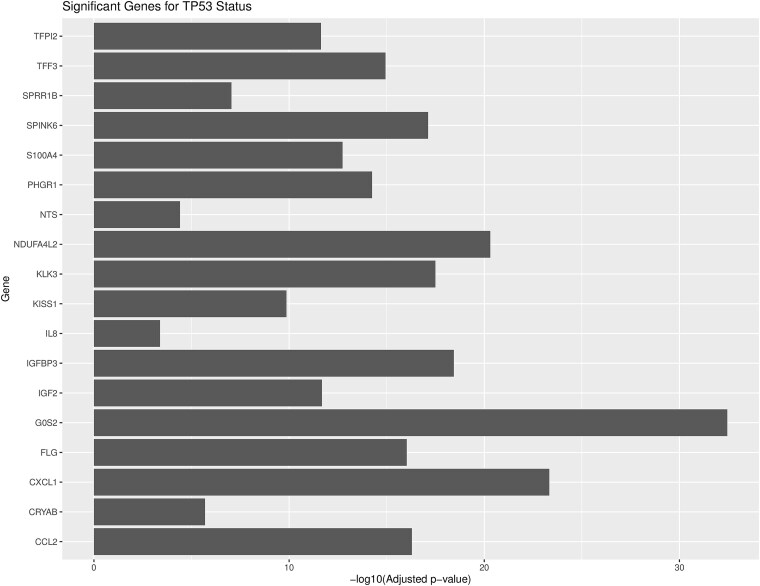
This figure displays the significant genes associated with TP53 status, highlighting their adjusted p-values. The analysis includes enhanced UMAP visualization and statistical testing between TP53_WT and TP53_MUT cell lines treated with Idasanutlin.

Statistical analysis of overall clustering patterns based on TP53 status: The statistical analysis includes enhanced UMAP visualization and statistical testing between TP53_WT and TP53_MUT cell lines treated with Idasanutlin. The adjusted p-values for the significant genes associated with TP53 statuses are shown in Fig. 3. Likewise, a volcano plot was generated to visualize differential gene expression between TP53_WT and TP53_MUT cell lines post-Idasanutlin treatment. This plot highlights significantly altered genes, providing mechanistic insights into the molecular responses associated with TP53 status (Fig. S1A). Additionally, Fig. S1B represents a heatmap for differentially expressed genes across different cell lines following the treatment of Idasanutlin, providing insights into its mechanism at the molecular level. Such representations can offer an understanding of how the mutational status of genes affects the drug response and contributes to developing resistance to the existing therapies. This information can be beneficial in catering personalized therapies for cancer patients.

The RNA-seq and scRNA-seq analysis reveals that wild-type TP53 is expressed in most cancers, which has a crucial role in governing DNA repair, angiogenesis, aging pathways, and apoptosis. Thus, rendering the overall p53 pathway a promising therapeutic target for developing medications that would benefit many patients. In the quest to find the interlinking regulators of the p53 pathway, we identified oncoprotein MDM2 as a potential target with druggable dependency [46, 47]. To assess the interaction of potential inhibitors with MDM2, we surveyed infected cancer cell lines expressing MDM2 and those subsequently treated with Idasanutlin (identified *via* our previous analysis). This treatment up-regulated MDM2 through a negative feedback mechanism triggered by higher p53 levels, as reported by Ding *et al.* in 2013 [34].

### Similarity search using machine learning

The above findings support the rationale for designing new molecules by combating resistance and increasing the efficacy of therapies in cancer treatment. Hence, we utilized Idasanutlin [34, 35, 48] as a query molecule for applying our ligand-based approach through conducting a similarity search-based screening. For this, we retrieved only bioactivity data from the ChEMBL database that are reported as pChEMBL values for tumor suppressor p53/oncoprotein MDM2 (CHEMBL1907611). From 1224 initial compounds, 443 compounds were available after data refinement for their pharmacokinetic parameters and Lipinski rule of five. The radar plot for all the compounds complying with the RO5 criteria is shown in Fig. S2.

### Calculate fingerprint descriptors

**Figure 4:**
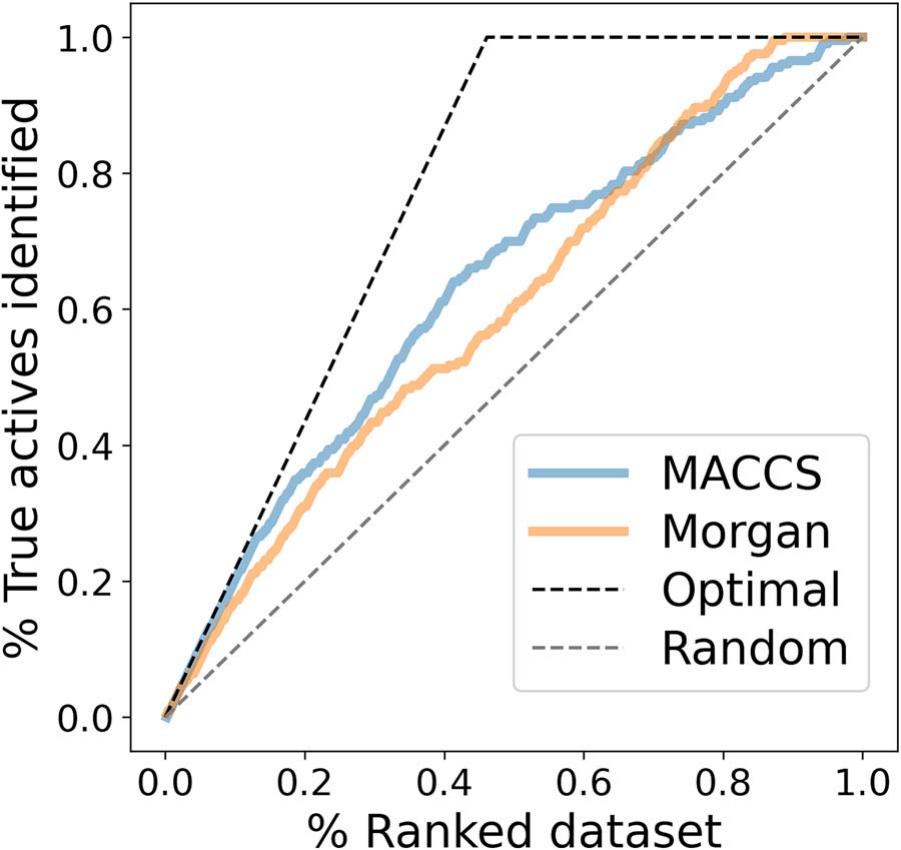
Enrichment plots with the pIC50 (log p-value) cut-off of 6.0 for MACCS and Morgan fingerprints to discriminate between active and inactive molecules.

Molecular encoding using descriptors/fingerprints aids in representing compounds quantitatively and this measure can then be used to assess for similarity with other compounds using different similarity matrices. In this work, Tanimoto and Dice similarity for all the filtered ChEMBL molecules, including the query molecule (Idasanutlin) was calculated using MACCS and Morgan fingerprints, as shown in Fig. S3. Further, from the initially filtered 443 compounds, we retrieved the most similar 248 compounds sharing a common chemical skeleton and plotted an enrichment plot using the pIC50 (log p-value) threshold of 6.0 to distinguish between the active and inactive molecules. The enrichment plot for MACCS and Morgan fingerprints (using the Tanimoto similarity) is shown in Fig. 4. Additionally, we calculated the experimental enrichment factor (EF) for 5% of the ranked dataset. The result showed that the experimental EF for 5% of the ranked dataset (tanimoto_maccs) was 10.3%, the experimental EF for 5% of the ranked dataset (tanimoto_morgan) was 8.4%, whereas the random EF for 5% of the ranked dataset was 5.0% and optimal EF for 5% of ranked dataset was 10.8%.

### Clustering of the molecules based on their fingerprint similarity

**Figure 5:**
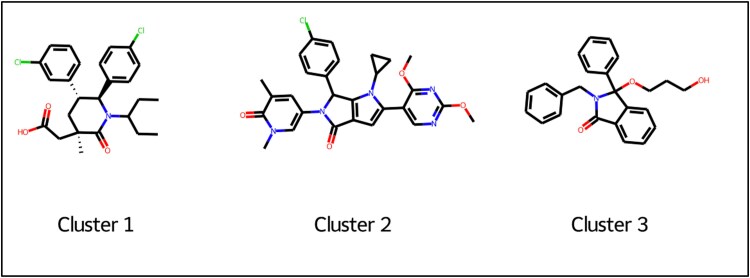
Plot of first three clusters where the number of molecules in the largest cluster (80), where the similarity between two random points in the same cluster was 0.90 while the similarity between two random points in the different cluster was 0.18.

**Figure 6:**
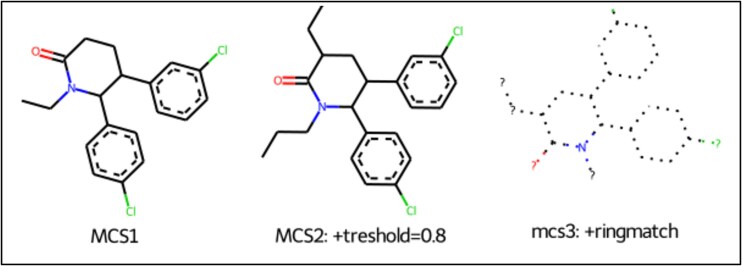
Substructure matched ring bonds where MCS2 contains 26 atoms and 28 bonds while MCS3 contains 24 atoms and 26 bonds.

After calculating the Tanimoto and Dice similarity for all the compounds, we aimed to cluster compounds by maximum common substructure (MCS). This ensures that the compounds that possess a specific chemical backbone and are responsible for corresponding their activity are included in a specific cluster in an iterative fashion. We performed MCS clustering on the previously retrieved ligands from CHEMBL against MDM2 using the FMCS algorithm [49], with different input parameters that align by the matched substructure for the same orientation, for the chosen 248 compounds (Fig. S5). As shown in Fig. 6, we observed the matched ring bonds, with MCS2 having 26 atoms and 28 bonds while MCS3 having 24 atoms and 26 bonds. As previously mentioned, and described in Fig. 6, we only kept molecules with pIC50 > 9 (*i.e.* IC50 < 1 nM) and performed our calculations on the highly active compounds that were chosen from the same three MCS variations (MCS1, MCS2, MCS3). The overall analysis can be interpreted from Fig. 4, Fig. 5, Fig. 6, Fig. S4, and Fig. S5.

### Ligand-based screening using machine learning

**Figure 7:**
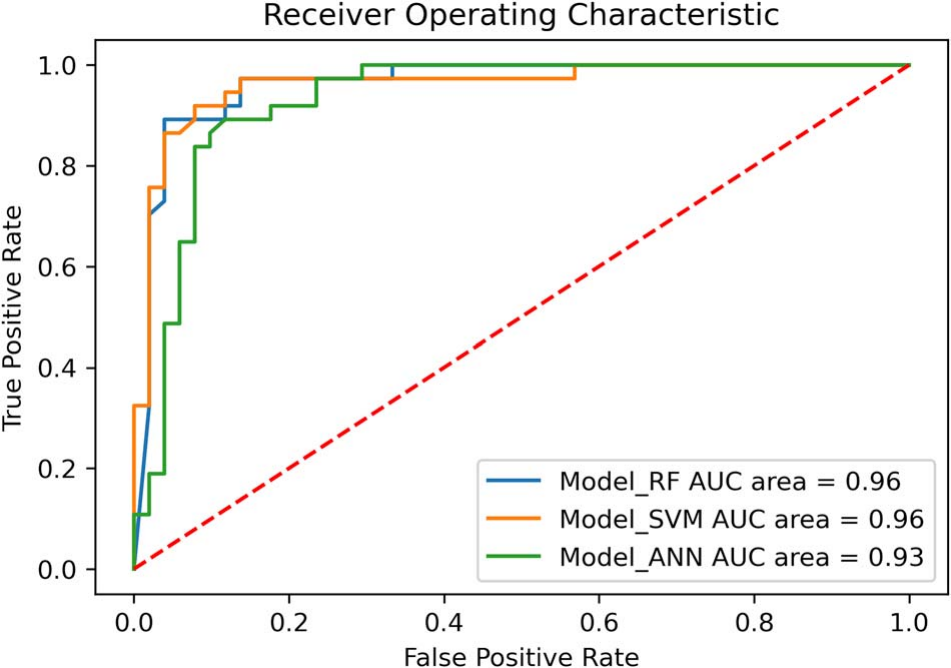
Comparison, assessment of the effectiveness, and evaluation of RF, SVM, and ANN model performances.

Further, we employed machine learning methods to classify compounds for their activity against MDM2. While the support vector classifier, with the set model parameter (with C = 1, gamma =0.1, the probability = True, and the SVM kernel = rbf), showed an accuracy of 0.89 and an AUC of 0.95, the accuracy of RF was 0.90 and the respective AUC was 0.95. Furthermore, a neural network classifier was used, with ANN hidden layer sizes set as 5 and 3. We found that the corresponding accuracy and AUC were 0.88 and 0.94 (details are shown in Fig. 7 and Table 1). We analyzed the cross-validation performance for compounds using the Morgan fingerprint for all three models. The results were comparable when we employed a Morgan fingerprint with a radius of 3, with the mean accuracy, sensitivity, specificity, and mean AUC for RF being 0.90, 0.91, 0.90, and 0.95. The results suggested that the performances for all models were great and RF was the best-performing model in our study. The RF model predicted 80 compounds as actives from the curated cluster of 248 compounds, that were further taken for molecular docking analysis.

### Molecular docking analysis

The tumor protein p53 or simply p53, a known oncoprotein suppressor is degraded by MDM2, an E3 ubiquitin ligase resulting in uncontrolled cell growth and tumor-like conditions [50, 51]. It has been reported that the hydrophobic cleft is formed on MDM2, offering the site for p53 to bind [34, 52–54]. The major interacting residues of the p53 domain which contribute to the binding with MDM2 are Phe19, Trp23, and Leu26 [54, 55]. Interrupting these interactions between the two proteins have paved the way to activate tumor suppression [55–57]. In our analysis, Hie96 and Lys94 are found to be the crucial residues present on the MDM2 site, by interfering with whom can disturb the MDM2-p53 complex system. The detailed workflow and analysis for the docking study has been provided in the supplementary data file.

**Figure 8:**
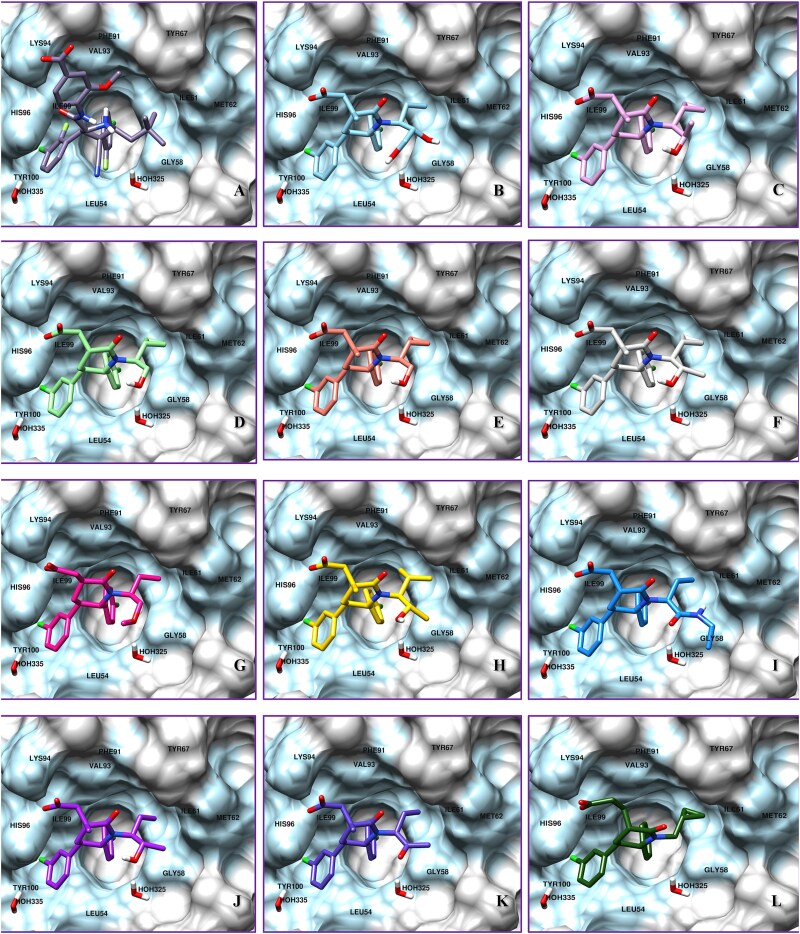
Docking poses of top 11 compounds around the p53 binding domain where A. depict Compound A (Idasanutlin, −6.406), B. depict Compound B (CHEMBL4110667, −10.241), C. depict Compound C (CHEMBL4111710, −10.129), D. depict Compound D (CHEMBL4115504, −10.084), E. depict Compound E (CHEMBL4108086, −10.071), F. depict Compound F (CHEMBL3975518, −10.057), G. depict Compound G (CHEMBL3966670, −9.870), H. depict Compound H (CHEMBL3983226, −9.673), I. depict Compound I (CHEMBL4113910, −9.666), J. depict Compound J (CHEMBL2059435, −9.644), K. depict Compound K (CHEMBL4110761, −9.632), L. depict Compound L (CHEMBL2177812, −9.566).

The predicted 80 compounds (out of 248) from machine learning were further subjected to molecular docking. The results were analyzed to compare for their corresponding docking scores, interactions, and orientation. Nine compounds have shown better affinity than AM-8553 and Idasanutlin in the docking analysis. Hence, we propose that our screened drugs can be potentially similar or even more active than both of the above standard compounds. The overall analysis of the docking study has been shown in the figure below Table 2 and Fig. 8.

**Table 2:** Top 11 Compounds based on the docking score, interactions, and orientations along with the occupancy of the receptor.

**Sr. No.**	**Compounds** **(Results)**	**Docking Score (XP)**	**Interactions**			**Figures**
			**Pi-Pi stacking**	**H-bonding**	**Salt-bridge**	
Ref	**AM-8553**	**−9.635**	Hie96	Hie96	Lys94	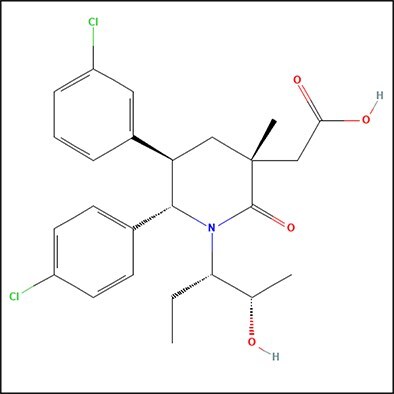
A	**Idasanutlin**	**−6.406**	Hie96	Hie96	Lys94	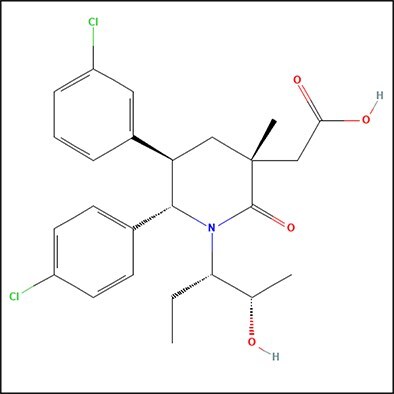
B	**CHEMBL4110667**	**−10.241**	Hie96	Hie96, Lys94	Lys94	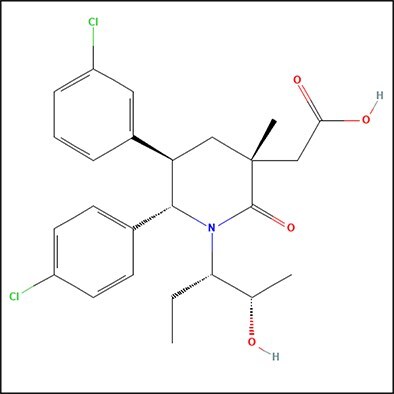
C	**CHEMBL4111710**	**−10.129**	Hie96	Hie96, Lys94	Lys94	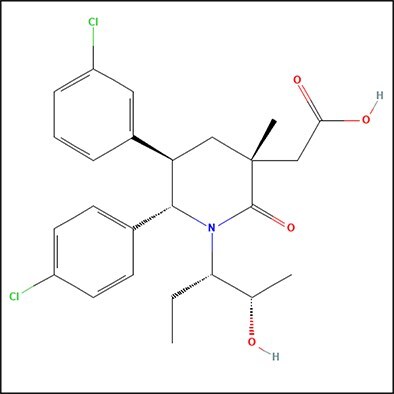
D	**CHEMBL4115504**	**−10.084**	Hie96	Hie96, Lys94	Lys94	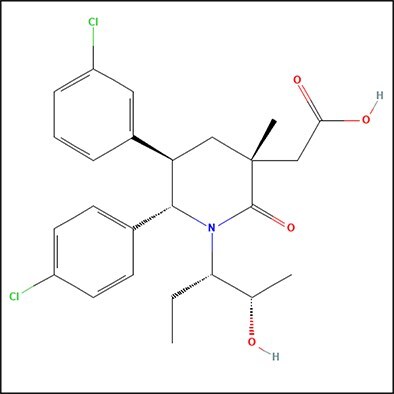
E	**CHEMBL4108086**	**−10.071**	Hie96	Hie96, Lys94	Lys94	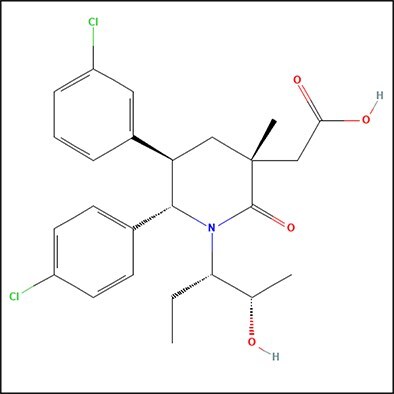
F	**CHEMBL3975518**	**−10.057**	Hie96	Hie96	Lys94	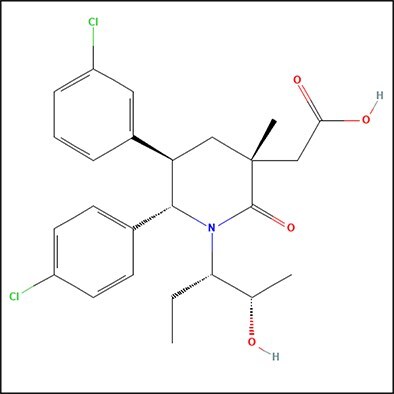
G	**CHEMBL3966670**	**−9.870**	Hie96	Hie96, Hie96#	Lys94	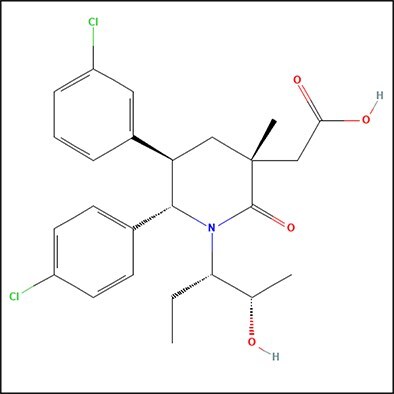
H	**CHEMBL3983226**	**−9.673**	Hie96	Hie96	Lys94	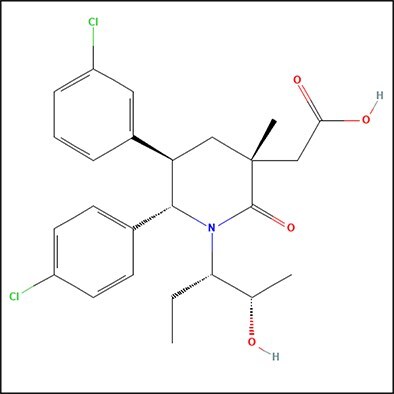
I	**CHEMBL4113910**	**−9.666**	Hie96	Hie96, Lys94, Gly58	Lys94	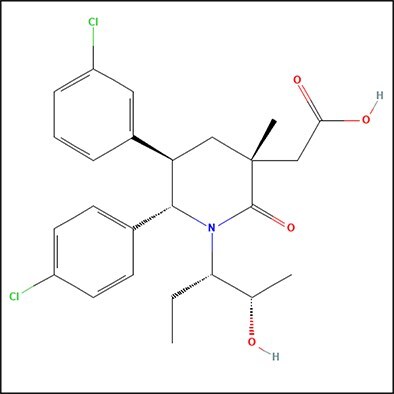
J	**CHEMBL2059435**	**−9.644**	Hie96	Hie96	Lys94	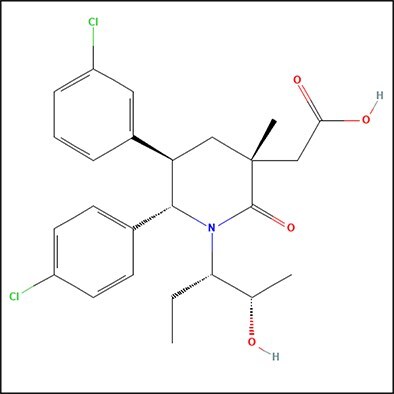
K	**CHEMBL4110761**	**−9.632**	Hie96	Hie96	Lys94	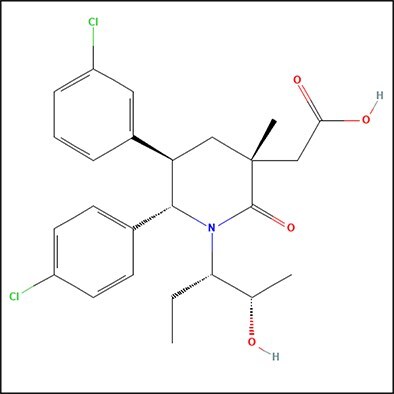
L	**CHEMBL2177812**	**−9.566**	Hie96	Hie96, Lys94	Lys94	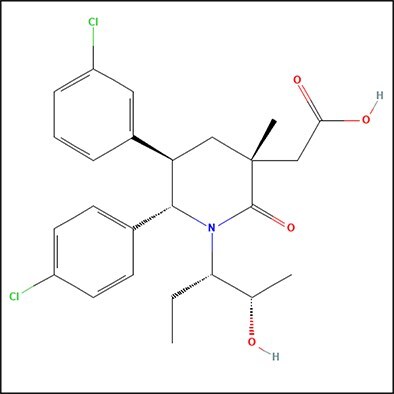

### Molecular dynamics simulation analysis

**Figure 9:**
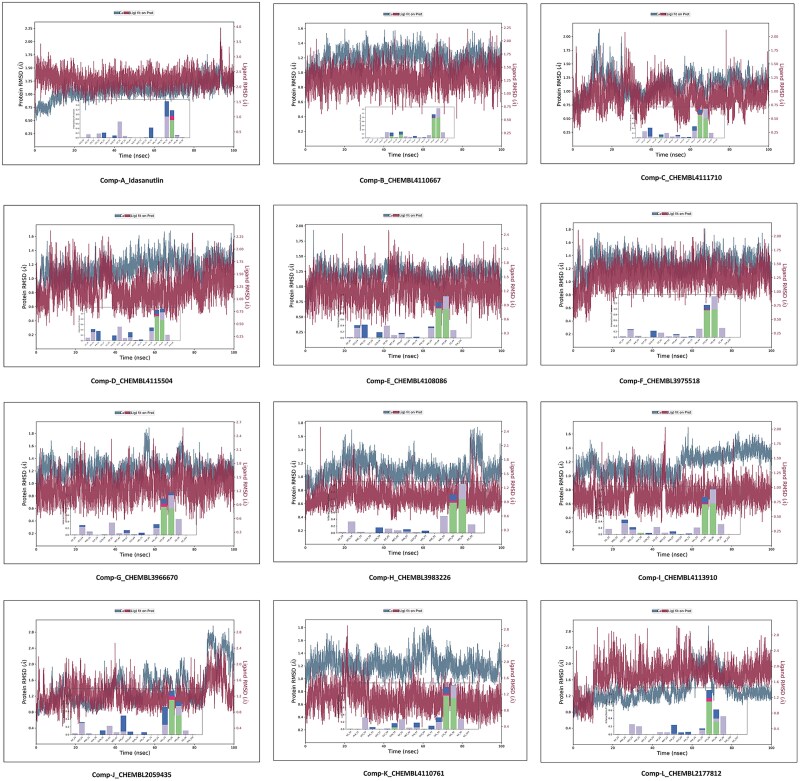
RMSD plots for Cα atoms of top 11 complexes along with the contribution of residues involved in the non-covalent interactions throughout the trajectory.

**Figure 10:**
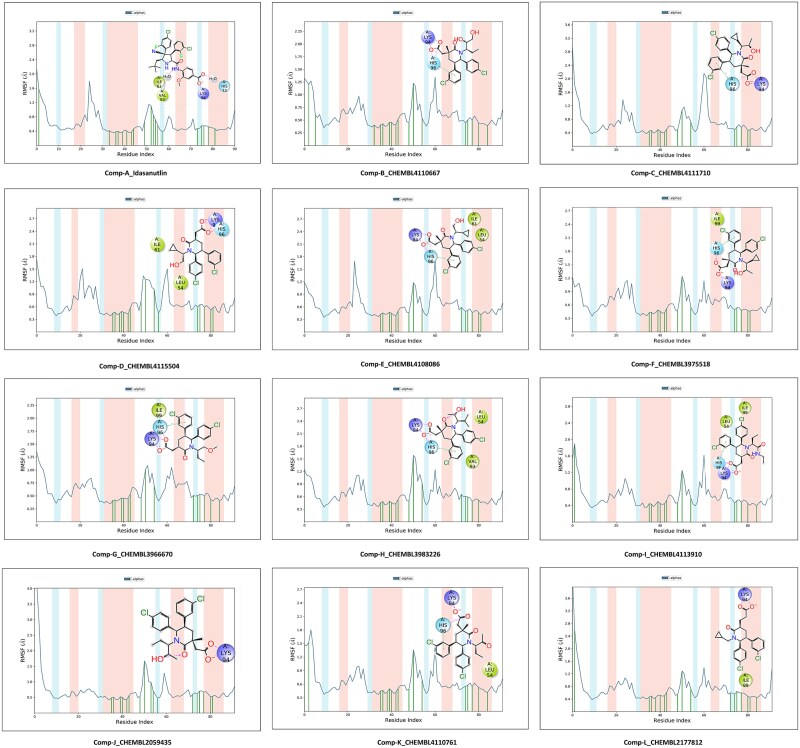
RMSF plots of the screened compounds along with the interactions of significant residues with corresponding molecules.

For evaluating our proposed study, MD Simulation was included into our process to confirm our docking analyses as well as to monitor the stabilities of the protein-ligand complexes within MDM2’s pocket. The time-path deviation from the initial structural orientation *i.e.* RMSD combined with RMSF for Cα atoms, was investigated for evaluating the stability of complex systems. Both were calculated post-analysis of MD simulation for 100 nanoseconds (ns), for all protein-ligand complexes (1300 ns) and were performed using the Desmond Schrodinger package. The RMSD plots are summarized in Fig. 9, and likewise, the RMSF plots are compiled in Fig. 10.

The RMSD and RMSF plots for complexes of AM-8553 (reference ligand) and compound A (Idasanutlin) were analyzed initially. In comparison to AM-8553, the Cα atoms of AM-8553-complex exhibited RMSD values between 1.2 and 1.7, whereas the complex of compound A showed comparatively lower RMSD values ranging from 0.6 to 1.5, depicted in Fig. S9. The stability for the complex of compound A has apparently increased, which is evident in the protein-ligand RMSD plot showing diminished fluctuations after the initial simulation as compared to the RMSD plot for AM-8553-complex, where fluctuations sustained throughout the simulation period. Hydrogen bonding, hydrophobic interactions, and ionic interactions were the basis for interaction fraction analysis.

The binding interaction fraction analysis compels the fact that Idasanutlin has a stronger binding affinity for MDM2 than AM-8553. So, the compounds screened after applying ML and similarity search approach can be potential candidates as MDM2 inhibitors. The RMSD and RMSF plots of the top 11 screened compounds also suggest that the complexes were comparatively stable over the period of simulation trajectory. Figure S7 and Fig. S8 represent the ligand torsion plots and important characteristics of top 11 screened compounds respectively, described in detailed in the supplementary file.

The major interactions (11 compounds) observed in our study were with Val93, Lys94, His96, and Ile99. By analyzing these results, we gain valuable understandings of the conformational changes, interactions, and structural properties of the ligands studied in our research. The RMSD values for screened compounds fell in the following average ranges; compound B: 1.25 to 1.50, compound C: 0.90 to 1.10, compound D: 1.30 to 1.50, compound E: 1.20 to 1.50, compound F: 1.20 to 1.40, compound G: 1.30 to 1.50, compound H: 1.00 to 1.20, compound I: 0.80 to 1.00, compound J: 1.40 to 1.60, compound K: 1.00 to 1.20, compound L: 1.80 to 2.00. The RMSF plot for the reference compound (AM-8553) showed slight fluctuations at the binding site of MDM2 as compared to the corresponding plot for Idasanutlin which conveys that the complex has been stabilized by the ligand. When the RMSF plots for our screened compounds were observed, it has come to our notice that the overall stability of the protein complex was more or less conserved in the whole simulation. The respective RMSF analysis of compounds C, E, F, G, I, J, and L proved that the complexes were comparatively more stable among the top 11 compounds. Further investigations revealed that compound J and compound L have shown better stability and lesser fluctuations in accordance with the reference compound (AM-8553) and in comparison, to compound A. Thus, the results of our docking study, followed by MD simulation analysis, ascertain the credibility of our study.

## Discussion

From the scRNA seq analysis done in our study, we observed the mutational pattern of p53 and its omnipresence in cancer subtypes. Owing to this fact from our analysis, we also found the evident role of the p53-MDM2 complex in various types of cancer progression from the literature [53, 55–57]. Being an E3 ubiquitin ligase, MDM2’s role of degrading p53 irrespective of its mutational pattern has resulted in tumor development and progression [50, 51, 58]. Many researchers have designed and synthesized compounds based on this to inhibit the degradation of p53 by MDM2 [52, 54, 59–61]. Idasanutlin was identified as a potential candidate from the scRNA seq analysis that showed its efficacy in wild-type and mutated p53 cell lines with co-expression of MDM2. It was developed by inducing modifications to a series of compounds enclosing a pyrrolidine scaffold and has shown some promising results clinically [34]. The nine drugs in the clinical trial insinuate the significance of inhibiting this MDM2-guided p53 degradation [62–69]. For our study, we have implemented a unique approach for identification of new scaffolds and substructures of the molecules that may disrupt the p53-MDM2 interaction, thereby inducing apoptosis and impeding tumor progression. We incorporated a combined approach of RNA-sequencing (RNA-seq) and single cell RNA-sequencing (scRNA-seq) analysis followed by machine learning-based screening, established on a similarity search scheme. The molecules we acquired as an output from the earlier approach were further analyzed using molecular docking and further subjected to molecular dynamics simulation.

As we know, MDM2 acts as a negative regulator of the TP53 cycle, and targeting MDM2 would be a better approach for druggability, as evident by the current inhibitors in the clinical trial. Hence, Idasanutlin was used as a template for employing a similarity search approach combined with machine learning for identifying potential lead molecules from the ChEMBL database based on Tanimoto and Dice similarities. Further, clusters of maximum common structures were calculated using the FMCS algorithm, generating the clusters of compounds which was selected for the next step of model building and validation. ML models like RF, SVM, and ANN were trained and validated by generating MACCS/Morgan fingerprints based on the enrichment factor. The 80 compounds were subjected to docking in the binding pocket for p53 on MDM2 protein (PDB ID: 4ERF). All 11 compounds were directed in the same orientation and had the same interactions with the bound ligand AM-8553. Out of 11 compounds, nine exhibited better docking scores than both Idasanutlin (docking score: −6.406) and AM-8553 (docking score: −9.635), insinuating that our approach using the RNA-seq data analysis along with machine learning methods has shown exciting results for potential small molecule inhibitors of the MDM2-p53 complex.

All 13 compounds (including the top 11 screened compounds, AM-8553 and Idasanutlin) were taken for MD simulations which provided significant insights into the interactions of the selected compounds against MDM2. The robust MD simulation of the top 13 compounds for the 1300 ns has provided significant insights into the interactions between the p53-MDM2 complex system and our screened molecules. Though the docking score of Idasanutlin was comparatively poor, the results from MD analysis showed better stability and lesser fluctuations than our reference compound (AM-8553; docking score: −9.635) which is shown in Fig. S10, providing in-depth significance of the contributing residues and demonstrating variation in interaction analysis of ligand-receptor complexes. Adding to this, compounds C, E, F, G, I, J, and L have shown better stability with the binding residues of the protein backed by the RMSD and RMSF plots for their complexes. The analysis revealed that compounds J and L exhibited comparatively lower fluctuations of the binding residues and were relatively more stable in their respective complexes than other compounds.

## Conclusion

Understanding the transcriptional effects of drugs on cancer cells is crucial in identifying their potential clinical application and molecular mechanisms. The dynamic interaction between MDM2 and p53 has revived the hope for designing novel drugs for anti-cancer therapy. In this study, researchers identified a robust response in TP53 cell lines, suggesting that Idasanutlin may be a promising candidate for cancer therapy. Furthermore, the study highlighted the importance of understanding mutational effects in different cancers pertaining to the prostate, ovary, and kidney. Integration of machine learning with the single-cell RNA-sequencing (scRNA-seq) and RNA-Seq data are emerging as promising avenues for predicting drug responses in cancer cells and enhancing cancer therapy. Furthermore, combining structure-based approaches with the above techniques can ensure robust screening of compounds for various cancers. These approaches allow for a more comprehensive understanding of the molecular alterations associated with cancer, making it possible to identify potential drugs for mutated cancer.

Additionally, the scRNA-seq analysis provided us with gene regulation with respect to the corresponding drug response. This study reflects the potential of Idasanutlin for cancer therapy, particularly in TP53 WT cell lines. The identification of robust transcriptional response signatures provides valuable insight into the underlying mechanisms of drug action and its potential clinical application. The sequential application of multiple-omics and scRNA-seq data analysis followed by machine learning-based virtual screening has been the main highlight of the study and signifies the rationale behind it. Finally, validating the outputs from ligand-based virtual screening with structure-based methods like molecular docking and dynamics shows the robustness of the study. This study suggests compounds showing potential for the design of future candidates as MDM2 inhibitors.

In context of future work, diverse type of multi-omics data can be incorporated with developing ML and DL algorithms which can unravel the complex mechanisms in the body and their inter-relationship with different tumor conditions. This knowledge can further help in identifying novel pathways for advancing the existing cancer therapy approaches. The combination of structure-based and ligand-based drug design methods can help to design new prototype for molecular scaffolds and help to fasten the drug discovery process. The scRNA-seq analysis showed that Idasanutlin exhibited greater efficacy in the case of both mutated and wild-type cell lines by down-regulating effector pathway genes of TP53 along with targeting different phases of cell growth. The next task was to find druggable cancer targets that would pave the way for new treatments while influencing the TP53 pathway. Identifying therapeutic targets through the analysis of multiple-omics data can also aid in developing novel and more effective treatments for cancer patients. With further research and refinement, the findings from this study could contribute to improving personalized medicine in cancer therapy in the upcoming future.

**Key Points**

- MDM2 inhibitors have shown assurance of therapeutic approach in cancer, as several potent and selective compounds have been developed in the recent years.
- Idasanutlin, a specific MDM2 inhibitor, was studied to understand its transcriptional effects on well-characterized cancer cell lines, aiming to elucidate its mechanism of action.
- The researchers observed a strong response in cancer cell lines treated with Idasanutlin, suggesting a significant impact on gene expression and indicating the drug’s potential efficacy.
- The study utilized machine learning techniques integrated on multi-omics and single-cell RNA-seq data to predict drug responses in cancer cells.
- The findings highlight the importance of identifying transcriptional response signatures for understanding the drug’s mechanisms of action and potential clinical application backed by molecular docking and dynamics analysis of the screened compounds from the ChEMBL database.

## Supplementary Material

SupplementaryData-07022025-MIS_elaf006

Table-S1_elaf006

Table_S2_elaf006

## Data Availability

All data are available upon reasonable request. This article contains all of the data generated and analyzed during this investigation. The Cancer Genome Atlas (TCGA) was used to download somatic mutation, gene expression, and clinical data *via* the Genomic Data Commons Data Portal (https://portal.gdc.cancer.gov/) and the R package ‘TCGAbiolinks’ on October, 2022; other cell line characteristics utilized in the investigation may be found on Figshare Dataset [24]. The crystal structure of MDM2 (PDB ID: 4ERF) was used to derive the initial complex structures. Python (v.3.7.11) script was used to generate ML model plots and grouped bar plots for evaluating model performance and similarity checking.

## Supplementary data

The Supplementary Information file included with the publication contains more supporting information, including graphics and the remaining specifics of this investigation (Supplementary data includes Fig. S1 to S13, Table S1 and Table S2). All relevant data and code used in this study can be accessed at our GitHub repository: (https://github.com/amanbioinfo/ML_based_Scaffold_Prediction).
