# Supplementary material for: Integrative machine learning approach for identification of new molecular scaffold and prediction of inhibition responses in cancer cells using multi-omics data
Source: Brief Funct Genomics. 2025 Apr 19;24:elaf006. doi: 10.1093/bfgp/elaf006 (PMC12008120; doi:10.1093/bfgp/elaf006)
Supplement: SupplementaryData-07022025-MIS_elaf006 [file supplementarydata-07022025-mis_elaf006.docx]

**Integrative Machine Learning Approach for Identification of new molecular scaffold and Prediction of Inhibition Responses in Cancer Cells Using Multi-Omics Data**

**Aman Chandra Kaushik^a^, Shubham Krushna Talware^a^, Mohammad Imran Siddiqi^a^**

^a^Division of Biochemistry and Structural Biology, CSIR-Central Drug Research Institute, Jankipuram Extension, Sitapur Road, Lucknow, Uttar Pradesh, 226031, India.

**Corresponding Author:** Mohammad Imran Siddiqi [mi_siddiqi@cdri.res.in](mailto:mi_siddiqi@cdri.res.in)

**Drug screening from single-cell RNA-Seq data**

The 24 different cancer cell lines used for this study are: DKMG_CENTRAL_NERVOUS_SYSTEM, BT549_BREAST, SQ1_LUNG, IALM_LUNG, CCFSTTG1_CENTRAL_NERVOUS_SYSTEM, NCIH2347_LUNG, RERFLCAD1_LUNG, BICR6_UPPER_AERODIGESTIVE_TRACT, LS1034_LARGE_INTESTINE, UMUC1_URINARY_TRACT, COV434_OVARY, SKMEL3_SKIN, LNCAPCLONEFGC_PROSTATE, BT474_BREAST, BICR31_UPPER_AERODIGESTIVE_TRACT, SH10TC_STOMACH, RCM1_LARGE_INTESTINE, SKMEL2_SKIN, RCC10RGB_KIDNEY, NCIH226_LUNG, TEN_ENDOMETRIUM, SNU1079_BILIARY_TRACT, CAOV3_OVARY, and COLO680N_OESOPHAGUS. For single-cell RNA sequencing, the cancer cell lines were dissociated into single cells, and the single cells were captured and sequenced using standard protocols. The responses to various chemical perturbation were observed across these cell lines.

The sequencing data was processed using the Cell Ranger pipeline to generate gene expression data. All scRNA-sequencing, drug sensitivity, and other cell line data are available on the Figshare Dataset. The single-cell data was analyzed using R packages, including Seurat, to identify cell types and to compare the transcriptional responses to drug perturbations. Finally, the cancer cell lines were used for in vitro drug screening, and the data on drug response was combined with data from RNA sequencing and single-cell RNA sequencing in order to computationally predict drug responses. These data were also used to explore the heterogeneity of drug response and cell line composition.

To identify the genes that were differentially expressed in response to Idasanutlin treatment, a differential expression analysis was performed. The volcano plot displayed a strong upregulation of TP53 effector pathway genes in TP53 wild-type cell lines. This effect was particularly evident in the volcano plot depicting the gene expression changes across TP53 wild-type cell lines in response to Idasanutlin treatment (Figure S1). For this and subsequent differential expression studies, effect size estimates and p-values were calculated. To illustrate the average log-fold change estimates for the top differentially expressed genes in each cell line, a heatmap was created. In contrast, TP53 mutant cell lines showed very minor alterations in gene expression in response to Idasanutlin therapy, as depicted in Figure S1.

**Single-cell expression profiles**

The PCA and UMAP analyses were carried out to explore the transcriptional response profiles of cell lines to varying treatment conditions. PCA was done on the log fold change profiles of 5000 genes with the maximum across-cell-line variance, with each gene mean-centered. Only cell lines with at least 10 cells in both the control and treatment conditions were included. The UMAP method, as implemented in the "Seurat" package, was then utilized to estimate the embedding of transcriptional response profiles. Response profiles were restricted to those with at least 10 cells per condition and at most 40 cells in total. The top 25 PCs were computed from the 5000 genes with the maximum variance across the selected profiles. UMAP was then run in this PC space, using cosine distance between samples, with an "n.neighbors" parameter of 15 and "min.dist" of 0.7. This investigation provided insights into the transcriptional response of cell lines against different treatment conditions and identified potential biomarkers of drug response.

For cell cycle analysis, the authors utilized the Seurat function CellCycleScoring, which employs gene lists specific to the S and G2M phases to classify cell cycle stages. An R function was used to evaluate how the proportion of cells in each phase varied under treatment and control conditions for each cell line. Aggregate scores, which depicted how each compound altered the cell cycle's composition, were calculated using weighted averages across cell lines based on variations in the proportion of cells in each phase. The measured drug sensitivity of the cell lines was used to calculate the weights, bounded between 0 and 1.


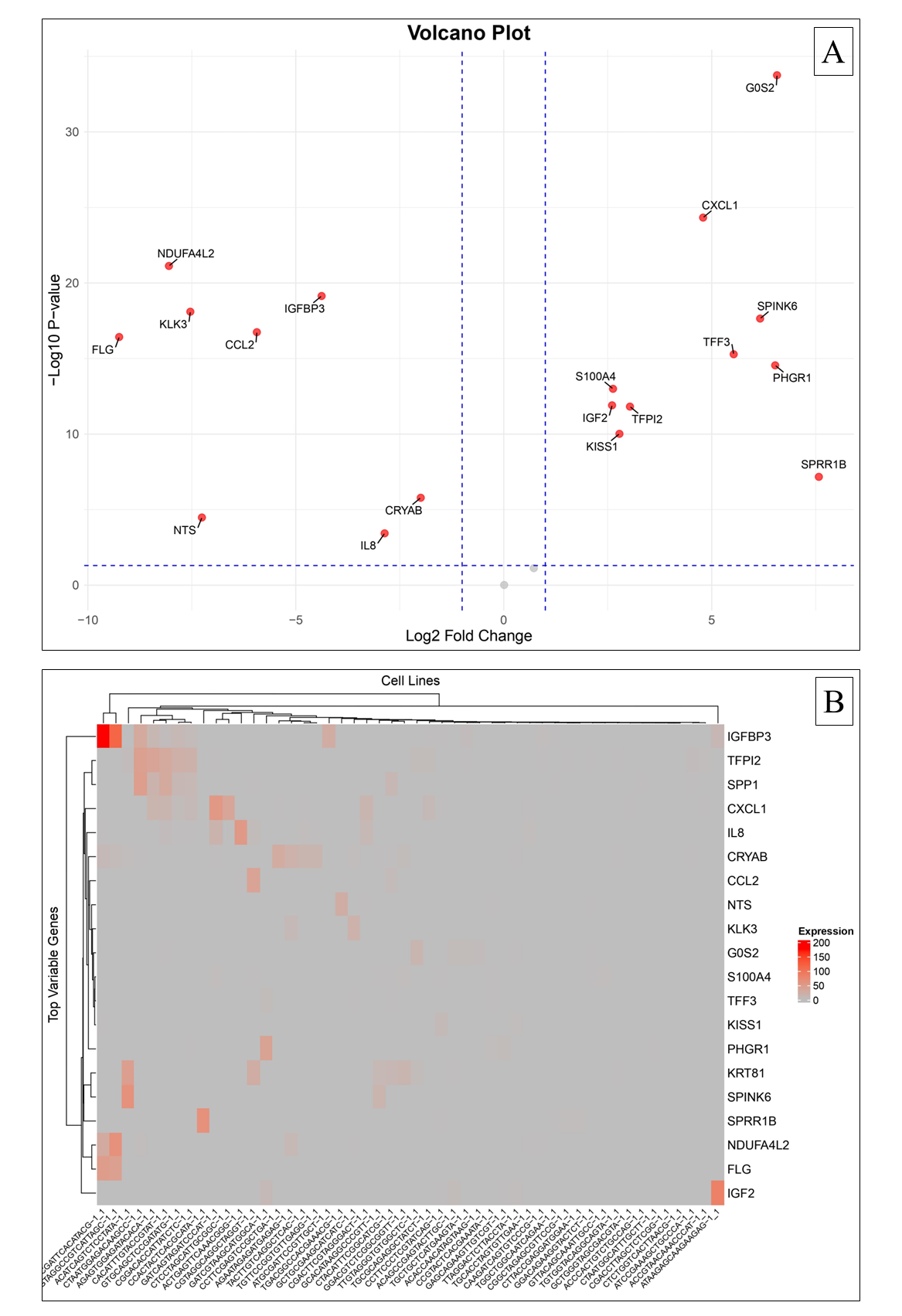


**Figure S1:** In response to Idasanutlin treatment: **A**. Volcano plot depicting significantly up-regulated/down-regulated genes of the TP53 effector pathway. **B**. Heatmap of the top 20 differentially expressed genes for a subset of 50 cell lines used in this work. The plot emphasizes the genes that are notably increased or decreased in reaction to the treatment of Idasanutlin offering an understanding of how it operates and its variance based on TP53 status.

**Similarity search for Tumour suppressor p53/oncoprotein MDM2 analysis using Machine Learning**

First, we converted the datatype of standard value from "object" to "float", then kept only entries with the standard unit (nM), followed by deleting duplicate molecules. Further, we labeled those compounds having IC50 values of less than 1000 nM (pIC50 >=6.0) as active; while those greater than 10,000 nM (pIC50 < 6.0) were referred to as inactive, and those with values between 1,000 and 10,000 nM were considered as intermediate respectively, for further evaluations. Then we did a Lipinski calculation to evaluate the drug-likeness of compounds, which is based on the pharmacokinetic profile or Absorption, Distribution, Metabolism, and Excretion (ADME). We found the total number of compounds in the unfiltered data set (1224), the total number of compounds in the filtered data set (443), and the total number of compounds not compliant with the Rule of five (784). We also did a PAINS (Pan Assay Interference Compounds) analysis and obtained 7 compounds identified as PAINS and 443 compounds as non-PAINS**.**

The statistical analysis for the dataset of compounds suggested that they fulfilled the rule of five (Figure S2), where the mean of molecular weight was 480.733 while the standard deviation was 65.692; for hydrogen bond acceptors, the mean was 4.500 and the standard deviation was 2.110, similarly for hydrogen bond donor, the mean was 1.086 and standard deviation was 0.884 and for logP value, the mean was 5.129 and the standard deviation was 0.899 respectively. We also did calculations for those who violated the rule of five. The mean of molecular weight was 596.724 while the standard deviation was 139.053, and the number of hydrogen bond acceptors showed a mean of 5.272 and standard deviation 2.441), for the number of hydrogen bond donors mean was 1.246 and the standard deviation 2.329) and similarly for logP value mean was 6.228 and the standard deviation was 1.168.

Then, we calculated MACCS and Morgan fingerprints for all compounds including controls. Tanimoto similarity and distance matrix were calculated for all compounds with respect to Idasanutlin (Figure S3). The clustering was performed for all compounds with respect to Idasanutlin. The resulting clusters were observed based on Tanimoto similarity and explained below (Figure S4).


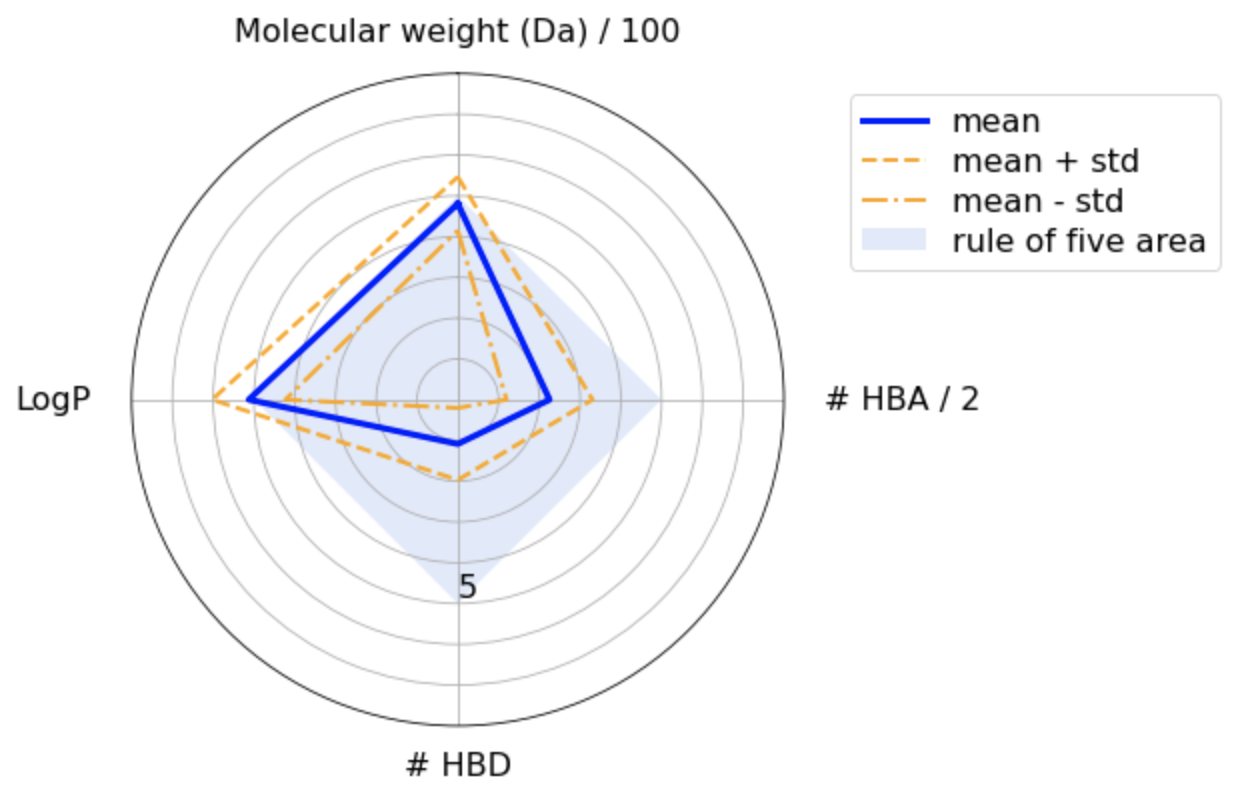


**Figure S2:** Radar plot providing a visual representation the dataset of compounds that fulfill the Rule of five (RO5) criteria. The shaded area depicts the boundary that a compound must lie in order to comply with RO5, whereas the orange line shows the spread (standard deviation) of the dataset indicating variability in the dataset, and the solid blue line shows mean values for each property and expected to lie within the shaded area. Since the blue line is entirely within the shaded area, it indicates that on average the compounds in the dataset adhere to RO5.


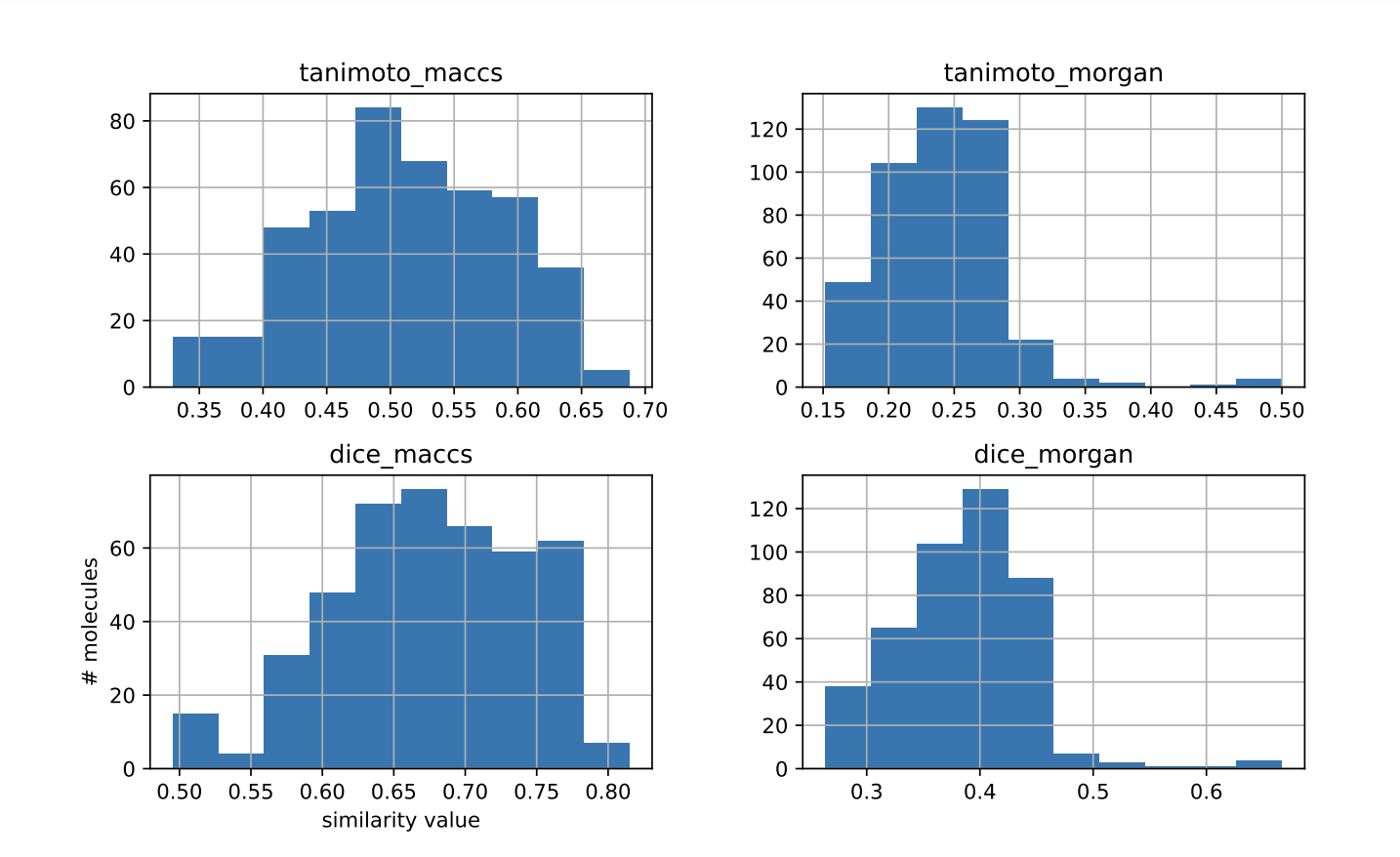


**Figure S3:** Distribution of similarity values for query molecule between (Idasanutlin) and all molecules using MACCS and Morgan. The plots representing the similarity are based on tanimoto and dice similarity indices with corresponding number of molecules (Y-axis) for similarity index corresponding (X-axis).


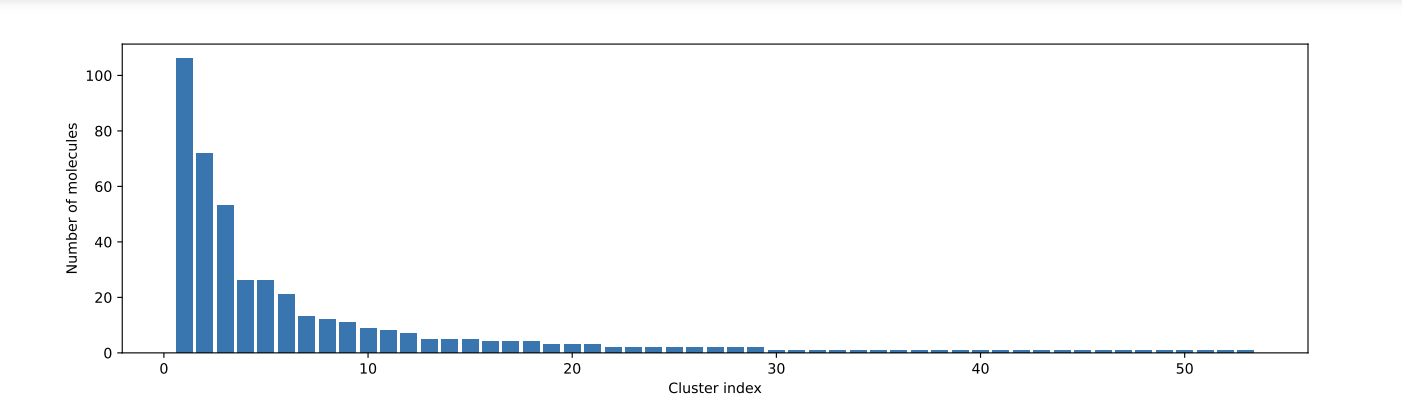


**Figure S4:** Clustering of the molecules based on their fingerprint similarity. The plot represents the indices of clusters formed (on the X-axis) and the corresponding molecules present in the respective clusters (on the Y-axis). Each cluster is formed on the basis of the most common chemical backbone that exist among molecules within that cluster and no cluster has any duplicate molecule. The cluster on the first index has largest number of molecules, and as we move from left to right, the number of molecules tends to decrease in the next sequential clusters.


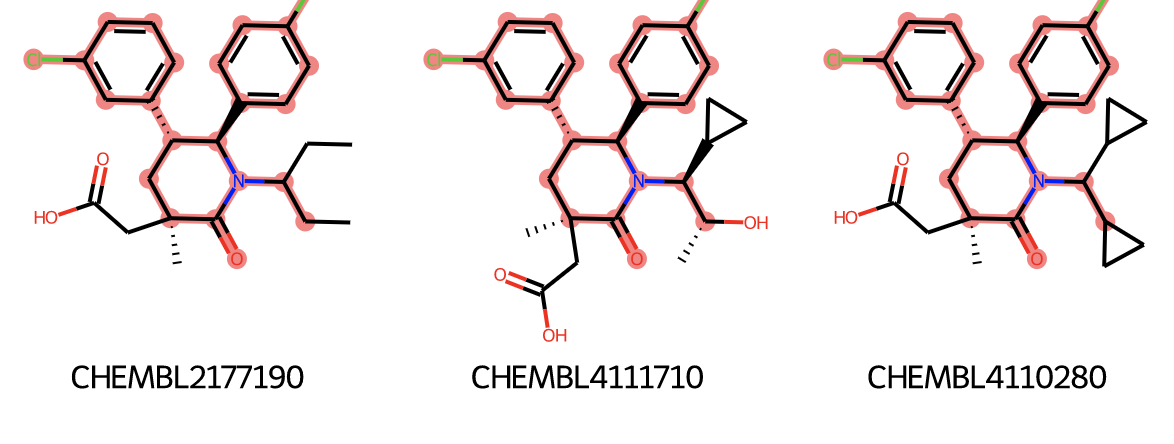


**Figure S5:** Calculation of maximum common substructure using FMCS algorithm. The highlighted structures in red, represents the primary scaffold that is present in majority of the compounds. It provides insights about important chemical backbone which can be explored for varying activity for different substitutions on the main scaffold.

**Molecular Docking Analysis**

Our machine learning was established on RG-7388 (Idasanutlin), based upon which we have retrieved the 80 molecules from the ChEMBL database using a machine learning based similarity search approach. Hence we also docked Idasanutlin (Compound A) into the pocket of AM-8553 (bound ligand, PDB ID: 4ERF). The docking score for which was -6.406, and the interactions reported were: i.e. Pi-pi stacking and hydrogen bonding interaction with Hie96 and salt bridge with Lys94. It has been observed that the interactions were conserved with respect to AM-8553. AM-8553 had shown a docking score of -9.635 which is shown in Figure S10. Moreover, these interactions around the MDM2 site were noted in all 11 compounds. Compound G has shown additional hydrogen bonding of the Cl atom with Hie96. Five compounds have shown an extra hydrogen bond with Lys94, named: Compounds B, C, D, E, I, and L, while preserving the AM-8553 interactions. Compound I has shown a unique

hydrogen bond with Gly58, along with other interactions mentioned above.


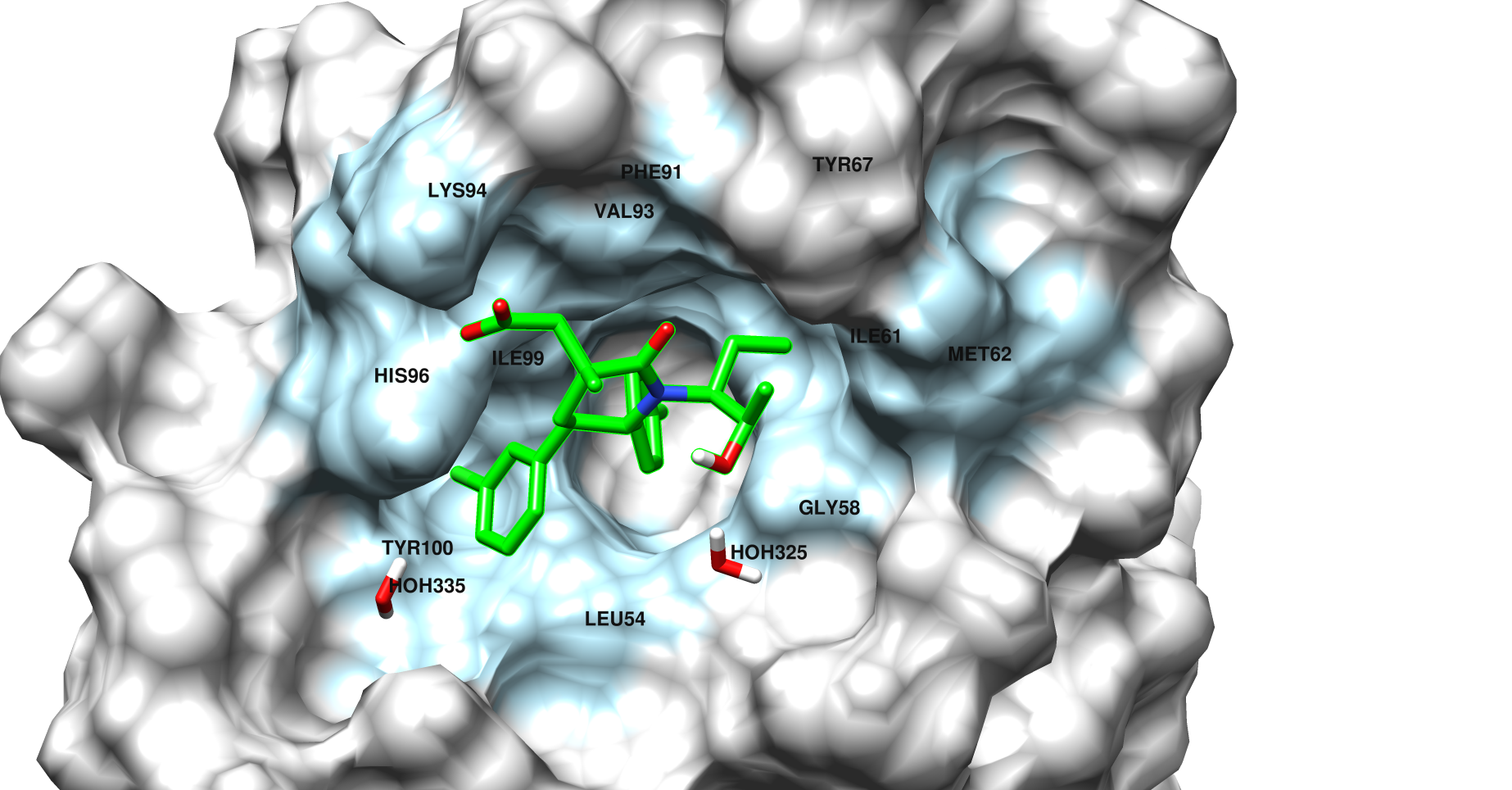


**Figure S6:** Docking pose of reference compound (AM-8553) around the p53 binding domain. The reported docking score was -9.635.

**Protocol for Molecular Dynamics Simulation Study**

The system was built in an orthorhombic simulation box using the simple point charge water (SPC) model and periodic boundary conditions (PBC). The neutralization of the generated system was achieved by incorporating counter ions and adjusting the SPC molecules’ geometry in the system. The SHAKE algorithm was used for neutralizing bond lengths of the heavy atom involving hydrogens, while electrostatic interactions were incorporated using the particle mesh Ewald (PME) method.

The simulation protocol comprised of several steps. Initially, the system, comprising the MDM2 structure with the top 13 compounds (compound A to compound M), was minimized by the steepest descent method with restraints on the solute for a maximum of 2,000 iterations. This was followed by the conjugate gradient method with a threshold energy of 50 Kcal/mol/Å. To establish equilibrium, a 10 picosecond (ps) simulation was executed at a temperature of 10K, on the non-hydrogen solute atoms in the NVT ensemble. Subsequently, a 100 ps MD simulation was conducted with restraints on the non-hydrogen solute atoms in the NPT ensemble at 10K temperature. Likewise, a 24 ps simulation was executed, allowing the system to relax devoid of restraints at 300K temperature in the NPT ensemble. Trajectories were recorded at intervals of 2.8 ps, with an energy recording interval of 1.2 ps.

**Molecular Dynamics Simulation Analysis**

The ligand torsions plot provides a comprehensive overview of the conformational evolution of all compounds along with controls throughout the simulation trajectory. The plot consists of a top panel showing a 2D schematic of the ligand, with rotatable bonds color-coded for easy identification. Each rotatable bond torsion is represented by a dial plot, where the conformational changes over time are visually displayed. Additionally, bar plots of the same color summarize the data from the dial plots, presenting the probability density of each torsion. In the MD simulation part, we present important findings related to the conformational evolution of the 11 compounds along with controls throughout the simulation trajectory (0.0 to 100 ns for each complex).

The ligand torsions plot of 11 compounds, along with controls, also incorporates information on the potential energy of the rotatable bonds, this is represented by the left Y-axis of the chart, where the values of the potential energy are shown alongside the histogram (Figure S11). The relationship between the torsional potential and the histogram provides information on the conformational strain experienced by the ligand in maintaining a bound conformation with the protein. Furthermore, Figure S12 summarizes various characteristics of the 11 screened compounds along with controls. Among these parameters, the ligand RMSD indicates the ligand’s root mean square deviation compared to the reference, usually the first frame of the simulation (t=0). The Radius of Gyration (rGyr) computes the "extendedness" of the ligand and corresponds to its principal moment of inertia. IntraHB represents the number of internal hydrogen bonds within the ligand molecule.


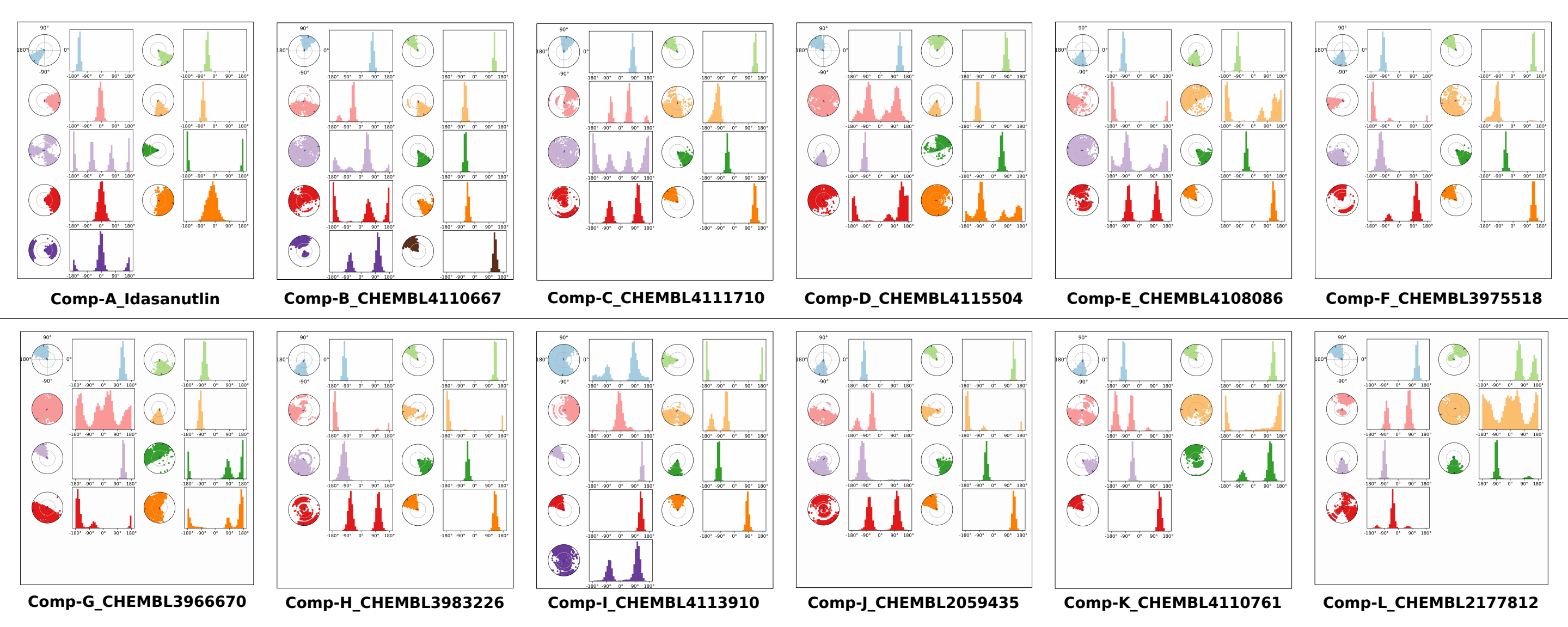


**Figure S7:** Ligand torsion plots summarizing the conformation evolutionary changes in the screened compounds throughout the simulation trajectory.


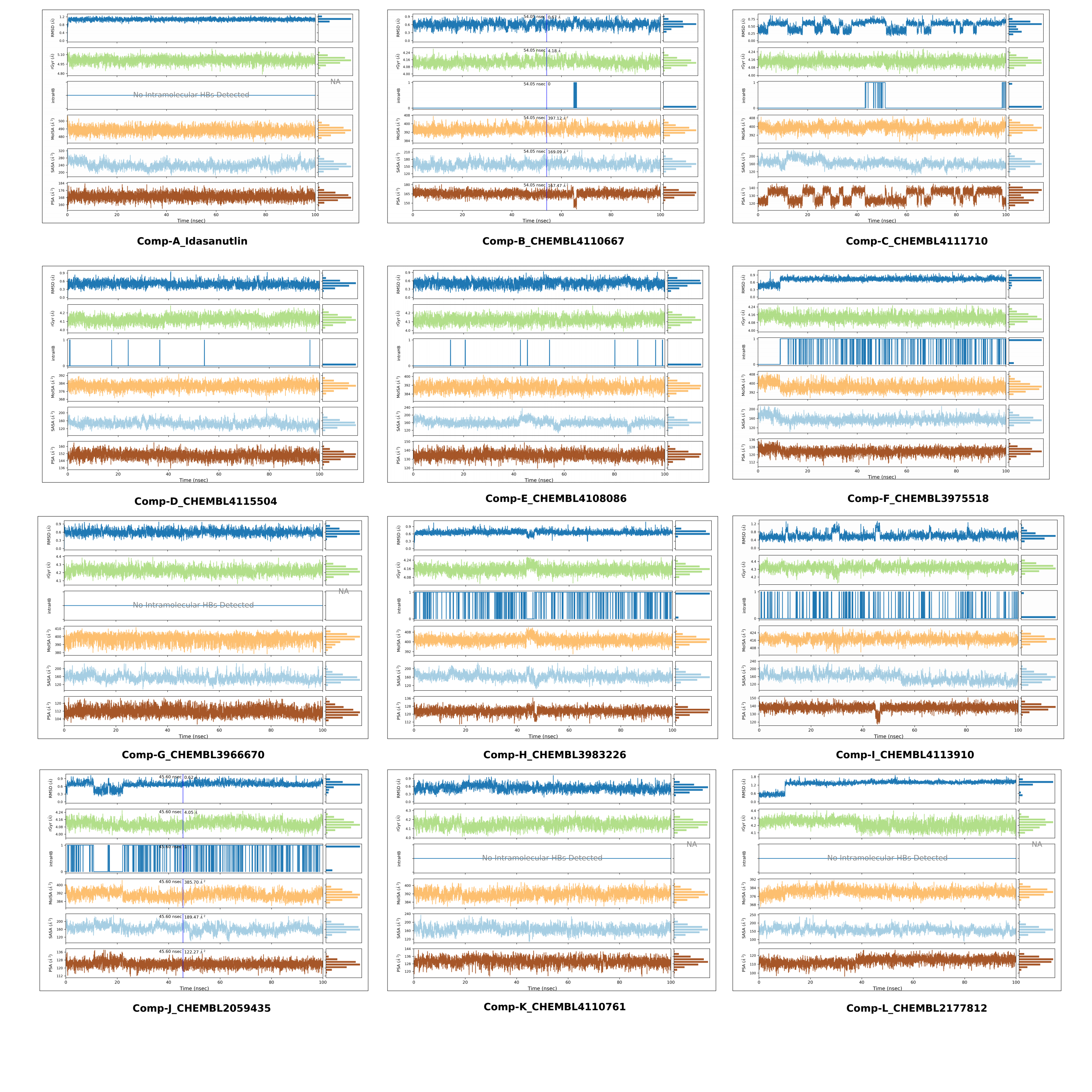
**Figure S8:** Plots summarizing various ligand properties including the RMSD, rGyr - Radius of Gyration, SASA - Solvent Accessible Surface Area, PSA – Polar Surface Area, IntraHB - Intramolecular Hydrogen Bonds, and MolSA - Molecular Surface Area, for the screened compounds.

**
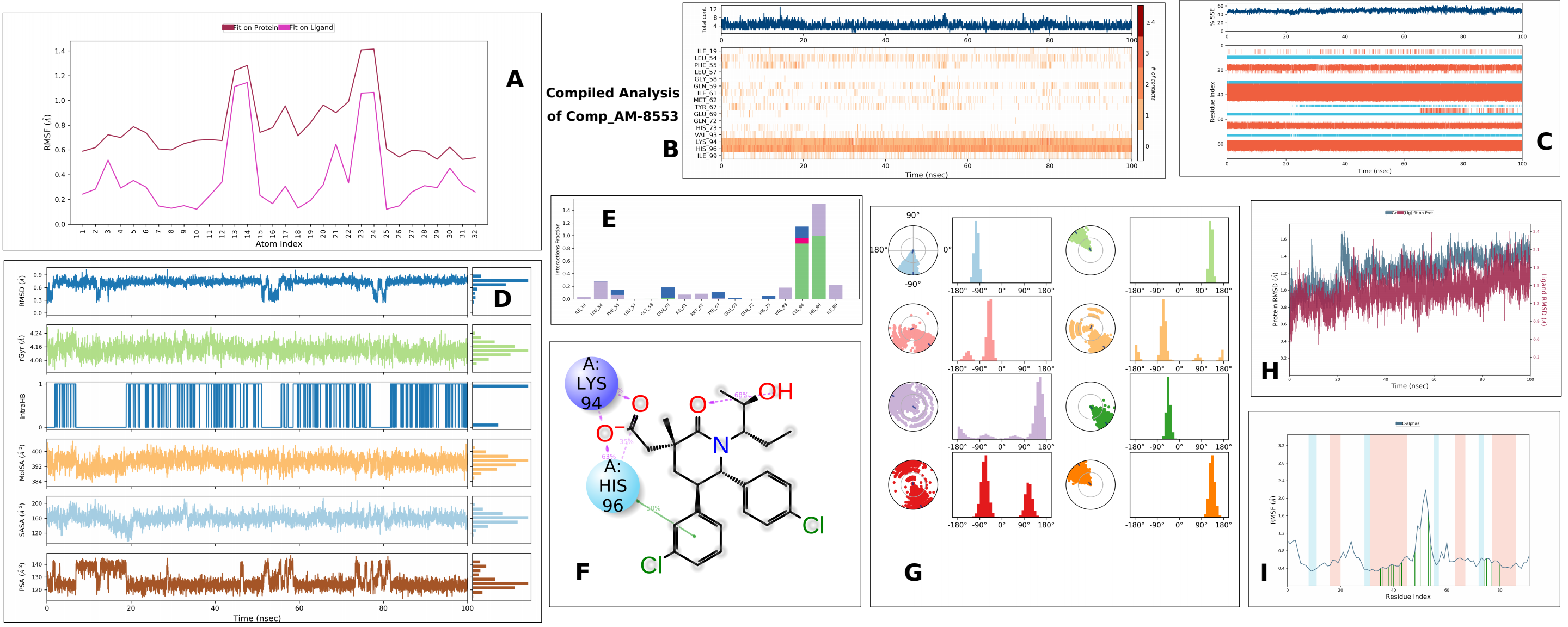
**

**Figure S9:** Figure showing all the compiled analysis for AM-8553 (reference compound).

**(A)** RMSF plot fit on the ligand of AM-8553-complex, **(B)** The interaction analysis during MD simulation, **(C)** depicts the SSE of AM-8553-complex, **(D)** depicts the PSA, SASA, MolSA, Intramolecular hydrogen bond interactions, Radius of gyration and RMSD of AM-8553-complex, **(E)** depicts interaction fraction, **(F)** depicts the ligand interaction diagram after MD simulation of AM-8553-complex, **(G)** depicts the torsion angle analysis of AM-8553-complex, **(H)** depicts the RMSD of AM-8553-complex ranged between 1.2 and 1.7 Å, not indicating any structural deviations. **(I)** Depicts the RMSF of AM-8553-complex ranged from 0.6 to 1.5 Å.

| Sr.No | gene | p_value.CRYAB | p_value.ND UFA4L2 | p_value. SPINK6 | p_value. KISS1 | p_value. NTS | p_value. S100A9 | p_value. TFF3 | p_value. KRT81 | p_value. KLK3 | p_value. PHGR1 | p_value. TFF1 | p_value. FLG | p_value. SPINK1 | p_value. IL8 | p_value. S100P | p_value. CCL2 | p_value. KRTAP2.3 | p_value. G0S2 | p_value. MLANA | p_value. IGF2 |
| --- | --- | --- | --- | --- | --- | --- | --- | --- | --- | --- | --- | --- | --- | --- | --- | --- | --- | --- | --- | --- | --- |
| 1 | CRYAB | -1.99562 | -8.04978 | 6.163416 | 2.780954 | -7.25708 | 6.778596 | 5.5283 | 0.009958 | -7.53529 | 6.525727 | 1.803461 | -9.24621 | 7.225017 | -2.86363 | -2.15154 | -5.93579 | -2.47576 | 6.569696 | 6.539159 | 2.604348 |
| 2 | NDUFA4L2 | 1.64E-06 | 7.42E-22 | 2.26E-18 | 9.60E-11 | 3.31E-05 | 4.12E-08 | 5.20E-16 | 0.971446 | 7.97E-19 | 2.82E-15 | 0.002221 | 3.75E-17 | 1.36E-13 | 0.000371 | 0.00076 | 1.80E-17 | 5.96E-06 | 1.77E-34 | 6.58E-32 | 1.25E-12 |
| 3 | SPINK6 | -1.99562 | -8.04978 | 6.163416 | 2.780954 | -7.25708 | 6.778596 | 5.5283 | 0.009958 | -7.53529 | 6.525727 | 1.803461 | -9.24621 | 7.225017 | -2.86363 | -2.15154 | -5.93579 | -2.47576 | 6.569696 | 6.539159 | 2.604348 |
| 4 | KISS1 | 1.64E-06 | 7.42E-22 | 2.26E-18 | 9.60E-11 | 3.31E-05 | 4.12E-08 | 5.20E-16 | 0.971446 | 7.97E-19 | 2.82E-15 | 0.002221 | 3.75E-17 | 1.36E-13 | 0.000371 | 0.00076 | 1.80E-17 | 5.96E-06 | 1.77E-34 | 6.58E-32 | 1.25E-12 |
| 5 | NTS | -1.99562 | -8.04978 | 6.163416 | 2.780954 | -7.25708 | 6.778596 | 5.5283 | 0.009958 | -7.53529 | 6.525727 | 1.803461 | -9.24621 | 7.225017 | -2.86363 | -2.15154 | -5.93579 | -2.47576 | 6.569696 | 6.539159 | 2.604348 |
| 6 | S100A9 | 1.64E-06 | 7.42E-22 | 2.26E-18 | 9.60E-11 | 3.31E-05 | 4.12E-08 | 5.20E-16 | 0.971446 | 7.97E-19 | 2.82E-15 | 0.002221 | 3.75E-17 | 1.36E-13 | 0.000371 | 0.00076 | 1.80E-17 | 5.96E-06 | 1.77E-34 | 6.58E-32 | 1.25E-12 |
| 7 | TFF3 | -1.99562 | -8.04978 | 6.163416 | 2.780954 | -7.25708 | 6.778596 | 5.5283 | 0.009958 | -7.53529 | 6.525727 | 1.803461 | -9.24621 | 7.225017 | -2.86363 | -2.15154 | -5.93579 | -2.47576 | 6.569696 | 6.539159 | 2.604348 |
| 8 | KRT81 | 1.64E-06 | 7.42E-22 | 2.26E-18 | 9.60E-11 | 3.31E-05 | 4.12E-08 | 5.20E-16 | 0.971446 | 7.97E-19 | 2.82E-15 | 0.002221 | 3.75E-17 | 1.36E-13 | 0.000371 | 0.00076 | 1.80E-17 | 5.96E-06 | 1.77E-34 | 6.58E-32 | 1.25E-12 |
| 9 | KLK3 | -1.99562 | -8.04978 | 6.163416 | 2.780954 | -7.25708 | 6.778596 | 5.5283 | 0.009958 | -7.53529 | 6.525727 | 1.803461 | -9.24621 | 7.225017 | -2.86363 | -2.15154 | -5.93579 | -2.47576 | 6.569696 | 6.539159 | 2.604348 |
| 10 | PHGR1 | 1.64E-06 | 7.42E-22 | 2.26E-18 | 9.60E-11 | 3.31E-05 | 4.12E-08 | 5.20E-16 | 0.971446 | 7.97E-19 | 2.82E-15 | 0.002221 | 3.75E-17 | 1.36E-13 | 0.000371 | 0.00076 | 1.80E-17 | 5.96E-06 | 1.77E-34 | 6.58E-32 | 1.25E-12 |
| 11 | TFF1 | -1.99562 | -8.04978 | 6.163416 | 2.780954 | -7.25708 | 6.778596 | 5.5283 | 0.009958 | -7.53529 | 6.525727 | 1.803461 | -9.24621 | 7.225017 | -2.86363 | -2.15154 | -5.93579 | -2.47576 | 6.569696 | 6.539159 | 2.604348 |
| 12 | FLG | 1.64E-06 | 7.42E-22 | 2.26E-18 | 9.60E-11 | 3.31E-05 | 4.12E-08 | 5.20E-16 | 0.971446 | 7.97E-19 | 2.82E-15 | 0.002221 | 3.75E-17 | 1.36E-13 | 0.000371 | 0.00076 | 1.80E-17 | 5.96E-06 | 1.77E-34 | 6.58E-32 | 1.25E-12 |
| 13 | SPINK1 | -1.99562 | -8.04978 | 6.163416 | 2.780954 | -7.25708 | 6.778596 | 5.5283 | 0.009958 | -7.53529 | 6.525727 | 1.803461 | -9.24621 | 7.225017 | -2.86363 | -2.15154 | -5.93579 | -2.47576 | 6.569696 | 6.539159 | 2.604348 |
| 14 | IL8 | 1.64E-06 | 7.42E-22 | 2.26E-18 | 9.60E-11 | 3.31E-05 | 4.12E-08 | 5.20E-16 | 0.971446 | 7.97E-19 | 2.82E-15 | 0.002221 | 3.75E-17 | 1.36E-13 | 0.000371 | 0.00076 | 1.80E-17 | 5.96E-06 | 1.77E-34 | 6.58E-32 | 1.25E-12 |
| 15 | S100P | -1.99562 | -8.04978 | 6.163416 | 2.780954 | -7.25708 | 6.778596 | 5.5283 | 0.009958 | -7.53529 | 6.525727 | 1.803461 | -9.24621 | 7.225017 | -2.86363 | -2.15154 | -5.93579 | -2.47576 | 6.569696 | 6.539159 | 2.604348 |
| 16 | CCL2 | 1.64E-06 | 7.42E-22 | 2.26E-18 | 9.60E-11 | 3.31E-05 | 4.12E-08 | 5.20E-16 | 0.971446 | 7.97E-19 | 2.82E-15 | 0.002221 | 3.75E-17 | 1.36E-13 | 0.000371 | 0.00076 | 1.80E-17 | 5.96E-06 | 1.77E-34 | 6.58E-32 | 1.25E-12 |
| 17 | KRTAP2-3 | -1.99562 | -8.04978 | 6.163416 | 2.780954 | -7.25708 | 6.778596 | 5.5283 | 0.009958 | -7.53529 | 6.525727 | 1.803461 | -9.24621 | 7.225017 | -2.86363 | -2.15154 | -5.93579 | -2.47576 | 6.569696 | 6.539159 | 2.604348 |
| 18 | G0S2 | 1.64E-06 | 7.42E-22 | 2.26E-18 | 9.60E-11 | 3.31E-05 | 4.12E-08 | 5.20E-16 | 0.971446 | 7.97E-19 | 2.82E-15 | 0.002221 | 3.75E-17 | 1.36E-13 | 0.000371 | 0.00076 | 1.80E-17 | 5.96E-06 | 1.77E-34 | 6.58E-32 | 1.25E-12 |
| 19 | MLANA | -1.99562 | -8.04978 | 6.163416 | 2.780954 | -7.25708 | 6.778596 | 5.5283 | 0.009958 | -7.53529 | 6.525727 | 1.803461 | -9.24621 | 7.225017 | -2.86363 | -2.15154 | -5.93579 | -2.47576 | 6.569696 | 6.539159 | 2.604348 |
| 20 | IGF2 | 1.64E-06 | 7.42E-22 | 2.26E-18 | 9.60E-11 | 3.31E-05 | 4.12E-08 | 5.20E-16 | 0.971446 | 7.97E-19 | 2.82E-15 | 0.002221 | 3.75E-17 | 1.36E-13 | 0.000371 | 0.00076 | 1.80E-17 | 5.96E-06 | 1.77E-34 | 6.58E-32 | 1.25E-12 |

**Table S1:** P-values for top 20 genes and their correlations.

| Sr.No | Gene | log2FoldChange | P_Value | P_adj |
| --- | --- | --- | --- | --- |
| 1 | CRYAB | -1.995617637 | 1.64E-06 | 2.34E-06 |
| 2 | NDUFA4L2 | -8.049775959 | 7.42E-22 | 4.95E-21 |
| 3 | SPINK6 | 6.163416215 | 2.26E-18 | 9.03E-18 |
| 4 | KISS1 | 2.780953607 | 9.60E-11 | 1.60E-10 |
| 5 | NTS | -7.257079212 | 3.31E-05 | 4.14E-05 |
| 6 | S100A9 | 6.778595929 | 4.12E-08 | 6.34E-08 |
| 7 | TFF3 | 5.528299875 | 5.20E-16 | 1.30E-15 |
| 8 | KRT81 | 0.009958304 | 9.71E-01 | 9.71E-01 |
| 9 | KLK3 | -7.535291249 | 7.97E-19 | 3.99E-18 |
| 10 | PHGR1 | 6.52572685 | 2.82E-15 | 6.26E-15 |
| 11 | TFF1 | 1.803461006 | 2.22E-03 | 2.34E-03 |
| 12 | FLG | -9.246208996 | 3.75E-17 | 1.07E-16 |
| 13 | SPINK1 | 7.22501712 | 1.36E-13 | 2.72E-13 |
| 14 | IL8 | -2.863633538 | 3.71E-04 | 4.36E-04 |
| 15 | S100P | -2.151536812 | 7.60E-04 | 8.45E-04 |
| 16 | CCL2 | -5.935786471 | 1.80E-17 | 6.00E-17 |
| 17 | KRTAP2-3 | -2.47576202 | 5.96E-06 | 7.95E-06 |
| 18 | G0S2 | 6.569695744 | 1.77E-34 | 3.54E-33 |
| 19 | MLANA | 6.539158811 | 6.58E-32 | 6.58E-31 |
| 20 | IGF2 | 2.604348155 | 1.25E-12 | 2.27E-12 |

**Table S2:** Adjusted p-values for top 20 genes.
