# Supplementary material for: Integrative machine learning approach for identification of new molecular scaffold and prediction of inhibition responses in cancer cells using multi-omics data
Source: Brief Funct Genomics. 2025 Apr 19;24:elaf006. doi: 10.1093/bfgp/elaf006 (PMC12008120; doi:10.1093/bfgp/elaf006)
Supplement: Table-S1_elaf006 [file table-s1_elaf006.docx]

| Sr.No | gene | p_value.CRYAB | p_value.ND UFA4L2 | p_value. SPINK6 | p_value. KISS1 | p_value. NTS | p_value. S100A9 | p_value. TFF3 | p_value. KRT81 | p_value. KLK3 | p_value. PHGR1 | p_value. TFF1 | p_value. FLG | p_value. SPINK1 | p_value. IL8 | p_value. S100P | p_value. CCL2 | p_value. KRTAP2.3 | p_value. G0S2 | p_value. MLANA | p_value. IGF2 |
| --- | --- | --- | --- | --- | --- | --- | --- | --- | --- | --- | --- | --- | --- | --- | --- | --- | --- | --- | --- | --- | --- |
| 1 | CRYAB | -1.99562 | -8.04978 | 6.163416 | 2.780954 | -7.25708 | 6.778596 | 5.5283 | 0.009958 | -7.53529 | 6.525727 | 1.803461 | -9.24621 | 7.225017 | -2.86363 | -2.15154 | -5.93579 | -2.47576 | 6.569696 | 6.539159 | 2.604348 |
| 2 | NDUFA4L2 | 1.64E-06 | 7.42E-22 | 2.26E-18 | 9.60E-11 | 3.31E-05 | 4.12E-08 | 5.20E-16 | 0.971446 | 7.97E-19 | 2.82E-15 | 0.002221 | 3.75E-17 | 1.36E-13 | 0.000371 | 0.00076 | 1.80E-17 | 5.96E-06 | 1.77E-34 | 6.58E-32 | 1.25E-12 |
| 3 | SPINK6 | -1.99562 | -8.04978 | 6.163416 | 2.780954 | -7.25708 | 6.778596 | 5.5283 | 0.009958 | -7.53529 | 6.525727 | 1.803461 | -9.24621 | 7.225017 | -2.86363 | -2.15154 | -5.93579 | -2.47576 | 6.569696 | 6.539159 | 2.604348 |
| 4 | KISS1 | 1.64E-06 | 7.42E-22 | 2.26E-18 | 9.60E-11 | 3.31E-05 | 4.12E-08 | 5.20E-16 | 0.971446 | 7.97E-19 | 2.82E-15 | 0.002221 | 3.75E-17 | 1.36E-13 | 0.000371 | 0.00076 | 1.80E-17 | 5.96E-06 | 1.77E-34 | 6.58E-32 | 1.25E-12 |
| 5 | NTS | -1.99562 | -8.04978 | 6.163416 | 2.780954 | -7.25708 | 6.778596 | 5.5283 | 0.009958 | -7.53529 | 6.525727 | 1.803461 | -9.24621 | 7.225017 | -2.86363 | -2.15154 | -5.93579 | -2.47576 | 6.569696 | 6.539159 | 2.604348 |
| 6 | S100A9 | 1.64E-06 | 7.42E-22 | 2.26E-18 | 9.60E-11 | 3.31E-05 | 4.12E-08 | 5.20E-16 | 0.971446 | 7.97E-19 | 2.82E-15 | 0.002221 | 3.75E-17 | 1.36E-13 | 0.000371 | 0.00076 | 1.80E-17 | 5.96E-06 | 1.77E-34 | 6.58E-32 | 1.25E-12 |
| 7 | TFF3 | -1.99562 | -8.04978 | 6.163416 | 2.780954 | -7.25708 | 6.778596 | 5.5283 | 0.009958 | -7.53529 | 6.525727 | 1.803461 | -9.24621 | 7.225017 | -2.86363 | -2.15154 | -5.93579 | -2.47576 | 6.569696 | 6.539159 | 2.604348 |
| 8 | KRT81 | 1.64E-06 | 7.42E-22 | 2.26E-18 | 9.60E-11 | 3.31E-05 | 4.12E-08 | 5.20E-16 | 0.971446 | 7.97E-19 | 2.82E-15 | 0.002221 | 3.75E-17 | 1.36E-13 | 0.000371 | 0.00076 | 1.80E-17 | 5.96E-06 | 1.77E-34 | 6.58E-32 | 1.25E-12 |
| 9 | KLK3 | -1.99562 | -8.04978 | 6.163416 | 2.780954 | -7.25708 | 6.778596 | 5.5283 | 0.009958 | -7.53529 | 6.525727 | 1.803461 | -9.24621 | 7.225017 | -2.86363 | -2.15154 | -5.93579 | -2.47576 | 6.569696 | 6.539159 | 2.604348 |
| 10 | PHGR1 | 1.64E-06 | 7.42E-22 | 2.26E-18 | 9.60E-11 | 3.31E-05 | 4.12E-08 | 5.20E-16 | 0.971446 | 7.97E-19 | 2.82E-15 | 0.002221 | 3.75E-17 | 1.36E-13 | 0.000371 | 0.00076 | 1.80E-17 | 5.96E-06 | 1.77E-34 | 6.58E-32 | 1.25E-12 |
| 11 | TFF1 | -1.99562 | -8.04978 | 6.163416 | 2.780954 | -7.25708 | 6.778596 | 5.5283 | 0.009958 | -7.53529 | 6.525727 | 1.803461 | -9.24621 | 7.225017 | -2.86363 | -2.15154 | -5.93579 | -2.47576 | 6.569696 | 6.539159 | 2.604348 |
| 12 | FLG | 1.64E-06 | 7.42E-22 | 2.26E-18 | 9.60E-11 | 3.31E-05 | 4.12E-08 | 5.20E-16 | 0.971446 | 7.97E-19 | 2.82E-15 | 0.002221 | 3.75E-17 | 1.36E-13 | 0.000371 | 0.00076 | 1.80E-17 | 5.96E-06 | 1.77E-34 | 6.58E-32 | 1.25E-12 |
| 13 | SPINK1 | -1.99562 | -8.04978 | 6.163416 | 2.780954 | -7.25708 | 6.778596 | 5.5283 | 0.009958 | -7.53529 | 6.525727 | 1.803461 | -9.24621 | 7.225017 | -2.86363 | -2.15154 | -5.93579 | -2.47576 | 6.569696 | 6.539159 | 2.604348 |
| 14 | IL8 | 1.64E-06 | 7.42E-22 | 2.26E-18 | 9.60E-11 | 3.31E-05 | 4.12E-08 | 5.20E-16 | 0.971446 | 7.97E-19 | 2.82E-15 | 0.002221 | 3.75E-17 | 1.36E-13 | 0.000371 | 0.00076 | 1.80E-17 | 5.96E-06 | 1.77E-34 | 6.58E-32 | 1.25E-12 |
| 15 | S100P | -1.99562 | -8.04978 | 6.163416 | 2.780954 | -7.25708 | 6.778596 | 5.5283 | 0.009958 | -7.53529 | 6.525727 | 1.803461 | -9.24621 | 7.225017 | -2.86363 | -2.15154 | -5.93579 | -2.47576 | 6.569696 | 6.539159 | 2.604348 |
| 16 | CCL2 | 1.64E-06 | 7.42E-22 | 2.26E-18 | 9.60E-11 | 3.31E-05 | 4.12E-08 | 5.20E-16 | 0.971446 | 7.97E-19 | 2.82E-15 | 0.002221 | 3.75E-17 | 1.36E-13 | 0.000371 | 0.00076 | 1.80E-17 | 5.96E-06 | 1.77E-34 | 6.58E-32 | 1.25E-12 |
| 17 | KRTAP2-3 | -1.99562 | -8.04978 | 6.163416 | 2.780954 | -7.25708 | 6.778596 | 5.5283 | 0.009958 | -7.53529 | 6.525727 | 1.803461 | -9.24621 | 7.225017 | -2.86363 | -2.15154 | -5.93579 | -2.47576 | 6.569696 | 6.539159 | 2.604348 |
| 18 | G0S2 | 1.64E-06 | 7.42E-22 | 2.26E-18 | 9.60E-11 | 3.31E-05 | 4.12E-08 | 5.20E-16 | 0.971446 | 7.97E-19 | 2.82E-15 | 0.002221 | 3.75E-17 | 1.36E-13 | 0.000371 | 0.00076 | 1.80E-17 | 5.96E-06 | 1.77E-34 | 6.58E-32 | 1.25E-12 |
| 19 | MLANA | -1.99562 | -8.04978 | 6.163416 | 2.780954 | -7.25708 | 6.778596 | 5.5283 | 0.009958 | -7.53529 | 6.525727 | 1.803461 | -9.24621 | 7.225017 | -2.86363 | -2.15154 | -5.93579 | -2.47576 | 6.569696 | 6.539159 | 2.604348 |
| 20 | IGF2 | 1.64E-06 | 7.42E-22 | 2.26E-18 | 9.60E-11 | 3.31E-05 | 4.12E-08 | 5.20E-16 | 0.971446 | 7.97E-19 | 2.82E-15 | 0.002221 | 3.75E-17 | 1.36E-13 | 0.000371 | 0.00076 | 1.80E-17 | 5.96E-06 | 1.77E-34 | 6.58E-32 | 1.25E-12 |
