# Supplementary material for: Integrative machine learning approach for identification of new molecular scaffold and prediction of inhibition responses in cancer cells using multi-omics data
Source: Brief Funct Genomics. 2025 Apr 19;24:elaf006. doi: 10.1093/bfgp/elaf006 (PMC12008120; doi:10.1093/bfgp/elaf006)
Supplement: Table_S2_elaf006 [file table_s2_elaf006.docx]

| Sr.No | Gene | log2FoldChange | P_Value | P_adj |
| --- | --- | --- | --- | --- |
| 1 | CRYAB | -1.995617637 | 1.64E-06 | 2.34E-06 |
| 2 | NDUFA4L2 | -8.049775959 | 7.42E-22 | 4.95E-21 |
| 3 | SPINK6 | 6.163416215 | 2.26E-18 | 9.03E-18 |
| 4 | KISS1 | 2.780953607 | 9.60E-11 | 1.60E-10 |
| 5 | NTS | -7.257079212 | 3.31E-05 | 4.14E-05 |
| 6 | S100A9 | 6.778595929 | 4.12E-08 | 6.34E-08 |
| 7 | TFF3 | 5.528299875 | 5.20E-16 | 1.30E-15 |
| 8 | KRT81 | 0.009958304 | 9.71E-01 | 9.71E-01 |
| 9 | KLK3 | -7.535291249 | 7.97E-19 | 3.99E-18 |
| 10 | PHGR1 | 6.52572685 | 2.82E-15 | 6.26E-15 |
| 11 | TFF1 | 1.803461006 | 2.22E-03 | 2.34E-03 |
| 12 | FLG | -9.246208996 | 3.75E-17 | 1.07E-16 |
| 13 | SPINK1 | 7.22501712 | 1.36E-13 | 2.72E-13 |
| 14 | IL8 | -2.863633538 | 3.71E-04 | 4.36E-04 |
| 15 | S100P | -2.151536812 | 7.60E-04 | 8.45E-04 |
| 16 | CCL2 | -5.935786471 | 1.80E-17 | 6.00E-17 |
| 17 | KRTAP2-3 | -2.47576202 | 5.96E-06 | 7.95E-06 |
| 18 | G0S2 | 6.569695744 | 1.77E-34 | 3.54E-33 |
| 19 | MLANA | 6.539158811 | 6.58E-32 | 6.58E-31 |
| 20 | IGF2 | 2.604348155 | 1.25E-12 | 2.27E-12 |

**Table S2:** Adjusted p-values for top 20 genes.
